# IL9 Polarizes Macrophages to M1 and Induces the Infiltration of Antitumor Immune Cells via MIP-1 and CXCR3 Chemokines

**DOI:** 10.1158/2767-9764.CRC-22-0246

**Published:** 2023-01-18

**Authors:** Van Anh Do-Thi, Sang Min Park, Song Mi Park, Hye Jin Jeong, Geunyoung Cho, Hyun-Jung An, Young Sang Kim, Hayyoung Lee, Jie-Oh Lee

**Affiliations:** 1 Institute of Membrane Proteins, POSTECH, Pohang, Republic of South Korea.; 2 Department of Life Sciences, POSTECH, Pohang, Republic of South Korea.; 3 Gyeongbuk Institute for Bio Industry, Andong-si, Gyeongbuk, Republic of South Korea.; 4 Department of Biochemistry, College of Natural Science, Chungnam National University, Daejeon, Republic of South Korea.; 5 Institute of Biotechnology, Chungnam National University, Daejeon, Republic of South Korea.

## Abstract

Tumor-associated macrophages (TAM) are involved in tumor progression, metastasis, and immunosuppression. Because TAMs are highly plastic and could alter their phenotypes to proinflammatory M1 in response to environmental stimuli, reeducating TAMs has emerged as a promising approach to overcoming the challenges of solid cancer treatment. This study investigated the effect of IL9 on macrophage M1 polarization and verified its antitumor potential to retrain TAMs and promote chemokine secretion. We demonstrated that IL9 stimulated macrophage proliferation and polarized them toward the proinflammatory M1 phenotype in an IFNγ-dependent manner. Tumor-localized IL9 also polarized TAMs toward M1 *in vivo* and made them release CCL3/4 and CXCL9/10 to recruit antitumor immune cells, including T and natural killer cells, into the tumor microenvironment. Furthermore, peritoneal treatment with recombinant IL9 delayed the growth of macrophage-enriched B16F10 melanoma and 4T1 breast cancer in syngeneic mice, although IL9 treatment did not reduce tumor growth in the absence of macrophage enrichment. These results demonstrate the efficacy of IL9 in macrophage polarization to trigger antitumor immunity.

**Significance:**

These findings clarified the effect of IL9 on macrophage M1 polarization and verified its antitumor potential through retraining TAMs and chemokine secretion.

## Introduction

Macrophages play critical roles in the innate immune response through the phagocytosis of and cytotoxic effects on foreign substances and serve as a bridge between the innate and acquired immune responses (1). In mice, activated macrophages can be classified into two different polarization states related to different stimuli, classically activated M1 and alternatively activated M2 subtypes (2). M1 macrophages, induced by Th1 cytokines such as IFNγ, IL12, and IL18, or the stimulation of Toll-like receptors, are proinflammatory and play a vital role in the host defense mechanism. In contrast, M2, induced by Th2 cytokines such as IL4, IL10, and IL13, are associated with anti-inflammatory responses and tissue remodeling. Notably, macrophages are highly plastic and can quickly alter their phenotype in response to signals in their local environment. In cancer, tumor-associated macrophages (TAM) are macrophages that infiltrate tumor tissue or settle in the microenvironment of solid tumors, and they often participate in tumor progression (2, 3). Although several studies mentioned that TAMs might exhibit the proinflammatory M1-like phenotype, recent studies suggested that general TAMs in aggressive tumors have the M2-like phenotype (4). M2 TAMs produce high amounts of anti-inflammatory cytokines, promote tumor development, and support metastasis. TAMs can also enhance tumor resistance to chemotherapy and radiotherapy (3). Accumulating evidence has shown that increased numbers of M2 TAMs were strongly associated with poor patient survival and aggressive characteristics in melanoma and breast cancer (5, 6). Hence, emerging therapeutic strategies targeting the functional reeducation of TAMs to M1-like mode attract more investigative attention.

IL9 is a cytokine member of the gamma-chain (γc) receptor family that includes IL2, IL4, IL7, IL15, and IL21. Its signaling is delivered via the IL9 receptor (IL9R) complex, which is comprised of the cytokine-specific IL9R α-chain and the common γc receptor (7). As a pleiotropic cytokine, IL9 can promote T-cell proliferation and differentiation, regulate the generation and activation of memory B cells, and induce the accumulation and functional maturation of mast cells (7). In the context of cancer, IL9 was shown to be a growth factor in most hematologic malignancies (8, 9). In solid tumors, IL9 promoted the growth of several types of pancreatic, colitis-associated, colon, or breast cancers by negatively regulating the antitumor function of T cells and promoting the immunosuppressive function of regulatory T cells (8). In contrast, IL9 can inhibit tumor progression directly by inhibiting the proliferation and migration of cancer cells or indirectly by activating innate and adaptive immunity to trigger antitumor responses (8, 10). High expression of IL9 in tumor tissues was related to prolonged survival of patients with colon carcinoma (11). Furthermore, we found that the number of tumor nodules in lungs intravenously injected with IL9-expressing B16F10 was 5-fold less than that of control groups and the percentages of T cells, natural killer (NK) cells, and M1 macrophages considerably increased in the lungs of the mice injected with IL9-expressing cells (12).

Therefore, we set out to conduct further experiments on the effect of IL9 on macrophage function. We found that IL9R was widely expressed on macrophages, and IL9-IL9R signaling stimulated the plasticity of macrophages toward the M1 phenotype and induced their proliferation. IL9 treatment also reeducated M2 macrophages and TAMs toward the M1 phenotype *in vitro* and *in vivo*. In macrophage-enriched cancers, IL9 polarizes TAMs to the M1 phenotype and triggers antitumor immunity by inducing chemotactic recruitment and the *in situ* accumulation of antitumor immune cells, including dendritic cells (DC), macrophages, T cells, and NK cells in CXCL9- and CCL3-dependent manners. Thus, we present for the first time that IL9 cooperated with macrophages to induce antitumor immunity through M1 polarization and chemokine induction.

## Materials and Methods

### Recombinant IL9 Expression and Purification

The recombinant mouse IL9 (rIL9) gene with a C-terminal hexahistidine and ALFA tag was cloned between the BamHI and NotI site in the pVL1393 baculovirus transfer vector. The recombinant baculovirus was generated by cotransfection of the BestBac 2.0v linearized baculovirus genome (Expression Systems) into Sf9 insect cells (Thermo Fisher Scientific). The rIL9 protein was produced by infecting High Five insect cells (Thermo Fisher Scientific) with the recombinant baculovirus. The secreted rIL9 protein was purified by Ni-Sepharose chromatography (GE Healthcare). After cleavage by thrombin to remove the hexahistidine and ALFA tag, the mIL9 protein was further purified by SP-Sepharose (GE Healthcare) cation-exchange chromatography and Superdex-200 gel filtration chromatography (GE Healthcare) equilibrated with a buffer containing 20 mmol/L Tris pH 8.0, 200 mmol/L NaCl, and 0.1 mmol/L phenylmethylsulfonyl fluoride. The protein sample buffer was changed into PBS (Gibco) using PD MiniTrap G-25 desalting columns (Cytiva). Endotoxin was removed from rIL9 using Pierce High-Capacity Endotoxin Removal Resin (Thermo Fisher Scientific), and the protein samples were sterilized by 0.2 μmol/L sterile membrane filtration. The protein concentration was determined using the Pierce BCA Protein Assay Kit (Thermo Fisher Scientific).

### Tumor Cell Lines and Mice

Female 6- to 8-week-old C57BL/6JBomTac and BALB/C mice were obtained from Daehan Biolink (Eumseong, Korea). All animal procedures were approved and guided by the Institutional Animal Care and Use Committee of Pohang University of Science and Technology (POSTECH-2020-0072). The murine melanoma B16F10 (80008) and RAW264.7 (40071), J774A.1 (40067), and P388D1 (10046) macrophage cell lines were purchased from Korean Cell Line Bank. The *Mycoplasma-*free cell lines were authenticated by the provider using DNA fingerprinting short tandem repeat profiling. Authenticated mammary carcinoma 4T1 cell lines were kindly provided by Prof. Young Sang Kim (Chungnam National University, Daejeon, Republic of South Korea). All cell lines were cultured in DMEM (Gibco) supplemented with 10% heat-inactivated FBS (Gibco), 2 mmol/L l-glutamine, and 100 U/mL penicillin plus 100 μg/mL streptomycin (1% Pen/Strep, Sigma-Aldrich) in a humidified 5% CO_2_ chamber at 37°C. Each cell line was expanded to produce frozen aliquots. After resuscitation, they were used for no more than 15 passages and no longer than 2 months. Cells were tested for *Mycoplasma* once a year. The IL9-secreting B16F10 cell line (B16F10-IL9) was generated as described previously (12). Briefly, B16F10 cells were transfected with the pcDNA3.1(+) vector containing the sequence of a secretory form of IL9 using Lipofectamine 2000 (Thermo Fisher Scientific). A B16F10 clone transfected with a mock vector was used as the control cells. G418 (Santa Cruz Biotechnology) was used at 1 mg/mL as a selective agent for the transfections.

### Bone Marrow–derived Macrophage Preparation

Bone marrow–derived cells (BMDC) were isolated from female C57BL/6J mice by flushing the femur and tibia with PBS. After removing the red blood cells, BMDCs were resuspended and cultured in DMEM containing 1% Pen/Strep, 20% FBS, and 1.25 μg/mL MCSF (315-02, Peprotech; Supplementary Fig. S1) at 37 °C. The MCSF-containing medium was changed after 3 days and the cells were cultured for another 3 to 4 days. Then, bone marrow–derived macrophages (BMDM) were harvested and characterized by the expression of macrophage surface markers, CD11b and F4/80 (Supplementary Fig. S1). The BMDMs were further sorted for the F4/80^+^PI^−^ population by FACS (S3e Cell Sorter, Bio-Rad). For the experimental setups, BMDMs were plated at a density of 15,000 cells/mm^2^ for 24 hours in MCSF-free DMEM. For qPCR analysis and the multiplex bead array experiments, BMDMs were treated with PBS, 100 ng/mL rIL9, or rIL9+2.5 μg/mL anti-IL9 for 48 hours. For the chemotaxis assay, BMDMs were treated with PBS, 100 ng/mL rIL9, or rIL9+1 μg/mL anti-IL9R for 48 hours. To study the repolarization of the macrophages, BMDMs were polarized into M2 macrophages by treatment with 20 ng/mL rIL4 (574304, BioLegend) for 24 hours. After 24 hours, IL4 was washed off, and the culture medium was replaced with medium containing 20 ng/mL rIL9 and incubated for an additional 48 hours.

### Antibodies

Anti-mouse IL9 antibody (504802, BioLegend), PE-conjugated anti-mouse CD129 (IL9R; 158703, BioLegend), anti-mouse IL9R mAb (MAB2134, R&D Systems), FITC-conjugated anti-hamster (Armenian) IgG (405502, BioLegend), anti-mouse CD16/CD302 (mouse BD Fc block, 553142, BD Biosciences), anti-mouse IFNγ antibody (513208, BioLegend), Armenian hamster anti-mouse CXCR3 (BE0249, InvivoMab, BioXcell), Armenian hamster control IgG (BE0091, InvivoMab), anti-CCL3/MIP-1α antibody (AF-450-NA, R&D Systems), anti-CCL4/MIP-1β antibody (AF-451-NA, R&D Systems), normal goat IgG control (AB-108-C, R&D Systems), anti-mouse CD3 (100301, BioLegend), APC/Cyanine7-conjugated anti-mouse CD45 (103116, BioLegend), PerCP/Cyanine5.5-conjugated anti-mouse/human CD11b (101228, BioLegend), APC-conjugated anti-mouse F4/80 (123116, BioLegend), FITC-conjugated anti-mouse CD80 (104705, BioLegend), FITC-conjugated anti-mouse CD86 (105005, BioLegend), FITC-conjugated anti-mouse CD206 (141703, BioLegend), FITC-conjugated anti-mouse CD11c (553801, BD Biosciences), APC-conjugated anti-mouse CD49b (103515, BioLegend), FITC-conjugated anti-mouse H-2Kb (116505, BioLegend), FITC-conjugated anti-mouse I-A/I-E (107605, BioLegend), FITC-conjugated anti-mouse TNFα (506303, BioLegend), PE-conjugated anti-human/mouse Arginase 1 (IC5868P, R&D Systems), PE-conjugated anti-STAT3 (678007, BioLegend), anti-STAT1 (abx012882, Abbexa), FITC-conjugated goat anti-rabbit IgG (554020, BD), APC/Cyanine7-conjugated anti-mouse CD3 (100222, BioLegend), FITC-conjugated anti-mouse CD4 (553729, BD Biosciences), PE-conjugated anti-mouse CD4 (553730, BD Biosciences), APC-conjugated anti-mouse CD8 (100711, BioLegend), APC/Cyanine7-conjugated anti-mouse CD3 (100222, BioLegend), PerCP/Cyanine5.5-conjugated anti-mouse/human granzyme B (372211, BioLegend), anti-pSTAT1 (Tyr701) antibody (9167, Cell Signaling Technology), anti-pSTAT3 (Tyr705) antibody (9145, Cell Signaling Technology), anti-pSTAT5 (Tyr694) antibody (ab32364, Abcam), anti-pSTAT6 (Tyr641) antibody (56554, Cell Signaling Technology), α‐Tubulin (T5168, Sigma-Aldrich), and horseradish peroxidase (HRP)-conjugated goat anti‐rabbit IgG (ab97051, Abcam).

### RNA Extraction and qPCR

RNA was extracted from 1 × 10^6^ BMDMs, RAW264.7 cells, and TAMs using the AccuPrep Universal RNA Extraction Kit according to the manufacturer's instructions (K-3140, Bioneer Corporation). The RNA concentration was determined by Ultrospec 8000 (GE Healthcare). Total RNA (1 μg) was reverse transcribed using AccuPower RT premix (Bioneer Corporation). For qPCR, RNA expression was analyzed on the QuantStudio 1 Real-Time PCR System (Thermo Fisher Scientific) using SFCgreen included in the 2X Real-time PCR Master Mix (DQ362-40h, BioFACT) at a concentration of 0.4 μL cDNA/sample. The primers for each of the genes are listed in Supplementary Table S1. Quantitative gene expression data were normalized to the expression levels of β-actin.

### ELISA

RAW 264.7 cells (1 × 10^5^/mL), J774A.1 cells (1 × 10^6^/mL), and BMDMs (1 × 10^6^/mL) were cultured in 12-well culture plates for 24, 48, and 72 hours. Then, IFNγ secreted into the culture supernatants was measured using an ELISA MAX Deluxe Set Mouse IFNγ kit (430804, BioLegend). To measure the level of IL9 secreted by the B16F10 transfectants, 2 × 10^5^/mL of B16F10-IL9 and B16F10-Mock cells were incubated in 24-well culture plates for 24 hours. Then, IL9 in the culture supernatants was measured using the ELISA MAX Deluxe Set Mouse IL9 kit (442704, BioLegend).

### FACS Analysis

The tissues were minced into small pieces and digested in DMEM containing 80 U/mL collagenase IV (17104-019, Gibco) and 80 U/mL DNase I (D5025, Sigma-Aldrich) at 37 °C for 30 minutes. The cells were subsequently filtered through a 40-μm cell strainer (93040, SPL).  After centrifuging, cells were blocked with 5 μg/mL of purified rat anti-mouse CD16/CD32 (553142, BD Biosciences) for 10 minutes at 4°C. For surface staining, the cells were stained with the appropriate antigen-specific antibodies conjugated to fluorophores or with an isotype control antibody for 1 hour at 4°C in the dark. All antibodies were diluted in staining buffer (1X PBS containing 0.02% sodium azide and 2% FBS). After washing off the unbound antibody with the staining buffer, the cells were analyzed using a flow cytometer (BD FACSCanto, BD Biosciences). To measure intracellular expression, the cells were cultured with a mixture of Brefeldin A (B5936, Merck, 1 μg/μL) and Monensin (M5273, Sigma-Aldrich, 3 μmol/L) for 4 hours. Then, the cells were fixed and permeabilized with BD Cytofix/Cytoperm Fixation and Permeabilization Solution (554722, BD Biosciences) according to the manufacturer's instructions. The permeabilized cells were incubated with an antibody diluted in staining buffer including 0.5% saponin for 1 hour at 4°C in the dark. For carboxyfluorescein succinimidyl ester (CFSE) staining, the cells were incubated with 2.5 μmol/L CFSE (423801, BioLegend) for 20 minutes. To distinguish dead cells, 10 μg/mL of propidium iodide (PI) was added to each sample just before the analysis.

### Cell Proliferation Assay

To analyze the proliferation of IL9-treated macrophages, 1 × 10^4^ RAW264.7, J774A.1, and BMDM cells were plated in 96-well plates. Cell proliferation was measured at the indicated timepoints after IL9 treatment using the 3-(4,5-dimethylthiazol-2-yl)-2,5-diphenyltetrazolium bromide (MTT) assay (M6494, Invitrogen). To compare the *in vitro* growth rate of the B16F10 transfectants, 1 × 10^4^ of B16F10-IL9 and B16F10-Mock cells were seeded in 96-well plates, and cell proliferation was analyzed by the MTT assay. The *in vitro* cytotoxicity of clodronate liposomes in macrophages was assessed as reported previously (13). Briefly, 1 × 10^5^ BMDMs and 1.5 × 10^4^ B16F10 cells were plated in 96-well plates. The cells were treated with either clodronate liposomes, control liposomes, or PBS at the indicated concentrations for 24 hours. Then, the cell viability was assessed by the MTT assay.

### Multiplex Bead Array Assay

Culture supernatants of IL9-treated BMDMs were used. To prepare cell lysates, the treated cells were incubated with 3 μmol/L monensin for the last 6 hours and harvested. The proinflammatory chemokine expression was determined using the LEGENDplex Mouse Proinflammatory Chemokine Panel (740007, BioLegend), following the manufacturer's instructions.

### Chemotaxis Assay

Chemotaxis assay was tested in 24-well Transwell chambers (5.0 μmol/L, polycarbonate membrane, Corning). BMDMs were treated with 100 ng/mL rIL9, rIL9 + 2.5 μg/mL anti-IL9R, or PBS for 48 hours. Then, the culture supernatants from the treated BMDMs were collected and placed in the lower chambers. Splenocytes were isolated from C57BL/6J mice. After red blood cell removal, 3 × 10^6^ cells were resuspended in 150 μL of DMEM and plated in the upper charmers. After assembling the chambers, the cells were incubated at 37°C for 2.5 hours. Then, the medium was aspirated from the upper chamber, and the nonmigrating cells in the upper chamber were scraped off with a cotton swab. The chambers were washed with PBS and then incubated with trypsin-ethylenediaminetetraacetic acid (EDTA) (Gibco) for an additional 5 minutes to collect all of the migrated cells. The cells were transferred to 5-mL U-bottom FACS tubes for further staining, and the number of migrated cell subsets was determined by FACS analysis. To analyze the chemotactic role of CCL3 secreted by IL9-treated macrophages, the culture supernatant from IL9-treated BMDMs was incubated with either 1.5 μg/mL of neutralizing anti-CCL3 or control IgG antibody for 30 minutes, and then loaded into the lower chamber for the chemotaxis assay. To examine the effect of CXCR3, splenocytes were pretreated with 3.8 μg/mL of a neutralizing anti-CXCR3 antibody or the isotype control for 30 minutes and then loaded into the upper chamber.

### Cytotoxicity Assay

Macrophage cytotoxic activity was measured using the MTT assay as described previously (14). To examine the cytotoxicity of macrophages against B16F10 melanoma, 1.5 × 10^6^ BMDMs were treated with either 100 ng/mL rIL9 or PBS for 48 hours and the IL9-containing medium was washed off and replaced with fresh culture medium. Then, the treated BMDMs were coincubated with 2 × 10^3^ target B16F10 cells at ratios of 2:1, 10:1, and 50:1 for 48 hours at 37°C. Simultaneously, the respective B16F10 target and effector BMDMs were also cultured. The activity was determined by measuring residual target cell viability using the MTT assay, and the percentages were calculated as follows:



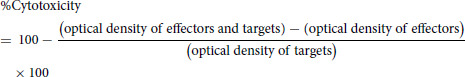



### Phagocytosis Assay

The macrophage phagocytosis of apoptotic cells has been described previously (15, 16). Apoptotic B16F10 tumor cells were derived by treatment with 10 μg/mL mitomycin C (CAS 50-07-7, Sigma-Aldrich) for 24 hours at 37°C. The efficacy of apoptotic induction was confirmed by annexing V (640905, BioLegend) and PI staining. After incubation, the tumor cells were washed twice with PBS and labeled with CFSE for 15 minutes. The CFSE-labeled cells were resuspended at 4 × 10^6^ cells/mL in complete DMEM. Then, the macrophages and apoptotic B16F10 cells were coincubated at a 1:2 ratio at 37°C for 1 to 4 hours. Flow cytometry was performed to obtain the percentage of CFSE^+^F4/80^+^ cells.

### Murine Tumor Models

For TAM studies, the coinoculation of BMDM and tumor cells has been described previously (17–19). With this system, the ratio of tumor cells to BMDMs varied from 1:1, 2:1, 4:1, to 5:1. We tested the ratios in the system using B16F10 and 4T1 cells, then we used a 2:1 ratio in the experiments. Briefly, 50 μL of a mixture of 2 × 10^5^ syngeneic BMDMs and 4 × 10^5^ B16F10 clone cells, either B16F10-IL9 or B16F10-Mock, were mixed with 50 μL of Matrigel (356230, BD Biosciences). Then, 100 μL of the final mixture was subcutaneously implanted into the flanks of C57BL/6J mice. The use of clodronate liposomes for the *in vivo* depletion of macrophages was described previously (13, 20, 21). In brief, macrophage depletion was achieved by the intraperitoneal injection of 200 μL of clodronate liposomes (Liposoma) every 3 days. As a control, a group of mice was injected intraperitoneally with control liposomes in PBS (Liposoma). For intraperitoneal treatment with the rIL9 model, BMDMs of syngeneic mice were mixed with B16F10 or 4T1 tumor cells at a 1:2 ratio in Matrigel, and subcutaneously implanted into the flanks of C57BL/6J or BALB/C mice, respectively (*n* = 3 for the dose-determining study, and *n* = 7 for the others). Tumor cells only were injected as controls. The tumor-bearing mice were treated intraperitoneally with rIL9 at a dose of 0.5 to 2 μg/mouse or PBS every 3 days. Tumor size was measured with a caliper, and tumor volume was calculated according to the following formula: 0.52 × *S*^2^ × *L*, where *L* is the length and *S* is the width of the tumor. Bodyweight was also monitored daily.

### Isolation of TAMs from Mouse Tumors

Fresh tumor tissue was minced into small pieces and digested in DMEM containing 80 U/mL collagenase IV and 80 U/mL DNase I at 37 °C for 30 minutes. The total cells were subsequently filtered through a 40-μm cell strainer and labeled with anti-F4/80 MicroBeads UltraPure (130-110-443, Miltenyi Biotec GmbH). The TAMs were sorted twice using MACS Columns (Miltenyi Biotec GmbH) and MACS Separators (Miltenyi Biotec GmbH) following the manufacturer's instructions.

### Hematoxylin and Eosin Histologic Staining

Tumor-bearing mice were sampled on day 12 after tumor implantation. The resected tumors were fixed in 4% paraformaldehyde for 11 hours. Tissue samples were mounted in paraffin blocks, and 4-μm sections were prepared. Sections were subjected to hematoxylin and eosin (H&E) staining. The morphologic changes in H&E-stained tissue, as well as tumor-infiltrating immune cells, were analyzed under an uplight microscope (OLYMPUS-BX43, OLYMPUS) at magnifications of 50× and 200×.

### Immunoblotting

RAW 264.7 cells were treated with 100 ng/mL rIL9 or PBS. At the indicated timepoints, total protein was extracted using 1X RIPA lysis buffer (20-188, Merck) containing a 1X solution of protease inhibitor cocktail (11836170001, Sigma-Aldrich) for 15 minutes. The protein concentration was determined by the BCA assay (23227, Thermo Fisher Scientific). Samples (20 μg/well) were resolved by 10% SDS‐PAGE. After transferring the proteins to polyvinylidene difluoride membranes using an electroblotter (AE-6677 HorizeBLOT, ATTO Corporation), the membranes were blocked with ProNA Phospho-Block Solution (TLP-115.1P, Translab) for 1 hour. Next, the membranes were incubated with primary antibodies overnight at 4°C and then incubated with secondary antibodies for 2 hours at room temperature. The blotted protein bands were detected with Immobilon Western Chemiluminescent HRP Substrate (WBKLS0500, Millipore) in the dark. The images were taken using an Ez Capture II machine (ATTO Corporation) and analyzed using ImageJ software.

### Statistical Analysis

All data are presented as the mean ± SEM (error bars). GraphPad Prism 7 (GraphPad) was used to perform one-way ANOVA and *t* tests to identify significant differences between groups (*, *P* < 0.05; **, *P* < 0.01; ***, *P* < 0.001; and ****, *P* < 0.0001). Survival data were analyzed by the Kaplan–Meier estimator using OriginPro 8.1 (OriginLab).

### Data Availability

The data generated in this study are available upon request from the corresponding authors.

## Results

### IL9-IL9R Signaling Promotes Macrophage Proliferation and IFNγ-dependent Polarization

**FIGURE 1:**
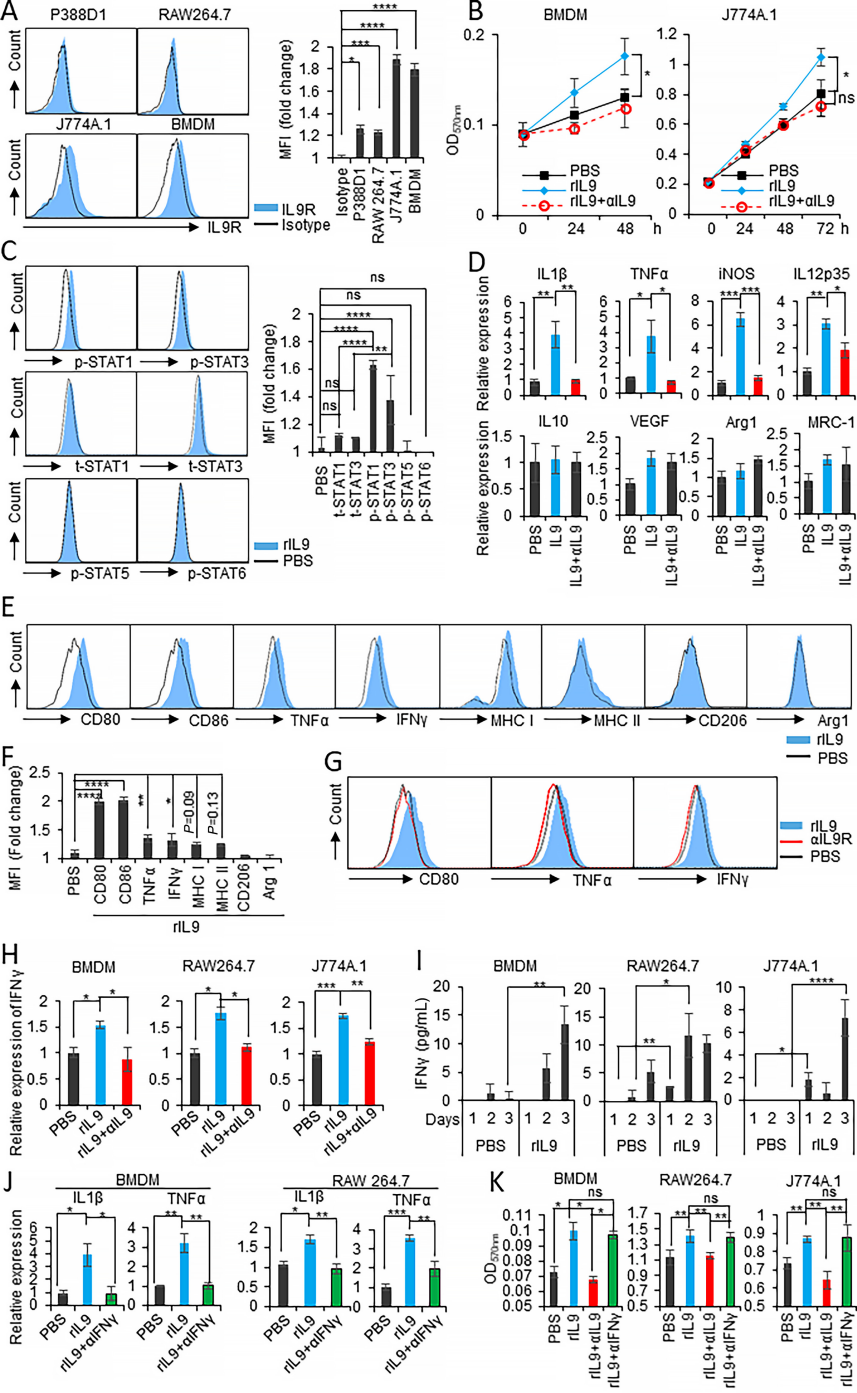
IL9-IL9R signaling promotes macrophage proliferation and M1 polarization *in vitro*. **A,** FACS analysis of the expression of IL9R in P388D1, RAW 264.7, and J774A.1 macrophages, and BMDMs using the anti-IL9R antibody. The relative mean fluorescence intensity (MFI) was calculated as follows: MFI of positive staining/MFI of isotype staining. **B,** MTT assay of BMDMs and J774A.1 macrophages stimulated with either 100 ng/mL rIL9, the mixture of rIL9+2.5 μg/mL anti-IL9, or PBS at the indicated timepoints. **C,** FACS analysis of total (t-) and phosphorylated (p-) STAT1, 3, 5, and 6 in BMDMs after 30 minutes of treatment with IL9. **D,** qPCR analysis of the mRNA expression of M1 markers (IL1β, TNFα, iNOS, and IL12p35) and M2 markers (IL10, VEGF, Arg1, and MRC-1) in BMDMs treated with either rIL9 or rIL9+anti-IL9 for 48 hours. **E,** FACS analysis of M1 markers (CD80, CD86, TNFα, IFNγ), the activation markers (MHC class I and II) and M2 markers (CD206 and Arg1) on BMDMs treated with either rIL9 or PBS for 48 hours using specific and isotype control antibodies. **F,** The relative MFI of each marker in E was calculated and the relative fold change compared with PBS was graphed. **G,** BMDMs was pretreated with anti-IL9R (5 μg/mL) or isotype control antibody for 30 minutes. Then, the pretreated BMDMs were treated with either IL9 or PBS for 48 hours. The expression level of M1 markers (CD80, TNFα, and IFNγ) were analyzed by FACS. **H,** BMDMs were treated with either rIL9 or rIL9+anti-IL9 for 48 hours. RAW 264.7 or J774A.1 macrophages were treated for 24 hours. Then, qPCR analysis of IFNγ mRNA in the treated macrophages was performed. **I,** The macrophages were treated with rIL9 or PBS for the indicated days, and IFNγ secretion into the culture medium by the treated macrophages was determined using an IFNγ-specific ELISA. **J,** qPCR analysis of IL1β and TNFα mRNA in BMDMs and RAW 264.7 macrophages treated with rIL9 or the mixture of rIL9+2.5 μg/mL anti-IFNγ. **K,** Proliferation assay (MTT assay) of BMDMs and RAW 264.7 or J774A.1 macrophages treated with rIL9, rIL9+anti-IL9, or rIL9+anti-IFNγ was performed 48 hours after treatment. *, *P* < 0.05; **, *P* < 0.01; ***, *P* < 0.001; and ****, *P* < 0.0001.

To examine the effects of IL9-IL9R signaling on macrophages, BMDMs and three macrophage cell lines, P388D1, RAW264.7, and J774A.1, were used. BMDMs were prepared as shown in Supplementary Fig. S1. IL9R was clearly expressed on the cell surface of all of these macrophages (Fig. 1A). Among them, BMDM and J774A.1 cells expressed higher levels of surface IL9R. Because IL9 is known as a growth factor of T cells and hematopoietic progenitor cells, the effect of IL9 on the proliferation of macrophages was tested. As shown in Fig. 1B, *in vitro* stimulation with rIL9 stimulated the proliferation of J774A.1 cells and BMDMs, and this growth-promoting effect was attenuated by the addition of anti-IL9 antibodies. IL9 mediates the growth-promoting effect in T cells by activating STAT1, STAT3, and STAT5 (22). Hence, we analyzed the phosphorylation of STAT transcription factors to verify the downstream signaling of IL9-IL9R in macrophages. Treatment with rIL9 induced STAT1 and STAT3 phosphorylation within 10 to 30 minutes in BMDMs as observed by FACS analysis of intracellular staining (Fig. 1C). However, no significant increase in p-STAT5 and p-STAT6 was observed in the macrophages. The rIL9 treatment did not significantly change total STAT1 (t-STAT1) and total STAT3 (t-STAT3) compared to PBS treatment. The phosphorylation of STAT1 and STAT3 was further confirmed in IL9-treated RAW264.7 cells using immunoblotting (Supplementary Fig. S2). Taken together, these results showed that IL9 treatment induced the phosphorylation of STAT1 and STAT3 in macrophages.

Macrophages can be activated to M1 or M2 subsets in response to signals in their local environment, and this plasticity is a critical property related to their functions (23). Therefore, we investigated the effect of IL9 stimulation on macrophage polarization. When nonactivated (M0) BMDMs were treated with rIL9, M1 markers, including IL1β, TNFα, Inducible nitric oxide synthase (iNOS), and IL12p35, were significantly induced at the mRNA level, but M2 markers, including IL10, VEGF, Arg1 (arginase 1), and MRC-1 (the CD206-coding gene), were not changed (Fig. 1D). The addition of anti-IL9 neutralizing antibodies attenuated the increase of expression of M1 markers but treatment with the isotype control antibody did not (Fig. 1D; Supplementary Fig. S3A). The M1 and M2 markers were also examined by FACS analysis. Consistent with the mRNA expression data, the representative cell surface markers of M1 macrophages (CD80 and CD86) were strongly induced by IL9 treatment (Fig. 1E and F). Other M1 markers (TNFα and IFNγ) and macrophage activation markers (MHC class I and MHC class II) were also induced by rIL9. However, there was no change in the expression of CD206 and Arg1. The M1 polarization effect of IL9 was prevented in BMDMs pretreated with anti-IL9R antibody (Fig. 1G). These results clearly showed that IL9 could induce M1 macrophage polarization. These effects were also found in RAW264.7 cells, showing that IL1β, TNFα, iNOS, and IL12p35 were significantly induced at the mRNA level and that the expression of IL10, VEGF, Arg1, and CCL17, was 2 to 4.5 times lower than in PBS-treated cells (Supplementary Fig. S3B). Similar to BMDM, the addition of anti-IL9 neutralizing antibody, but not isotype control antibody, abolished the effect (Supplementary Fig. S3C).

Because IFNγ is responsible for one crucial axis of M1 polarization in both human and murine macrophages (24), we assessed whether IL9-treated macrophages could be a source of IFNγ. Consistent with the FACS analysis results in Fig. 1E, IFNγ mRNA expression was also enhanced 1.5-fold in rIL9-treated macrophages and was attenuated by adding an anti-IL9 antibody (Fig. 1H). To further verify IFNγ secretion from IL9-treated macrophages, the level of IFNγ was analyzed in the culture supernatants from BMDMs, RAW264.7, and J774A.1 cells, and a detectable amount was found upon rIL9 treatment. The amount increased in a time-dependent manner to reach about 10 pg/mL at 72 hours after rIL9 treatment (Fig. 1I). We next analyzed whether IL9-induced M1 polarization was IFNγ dependent. The addition of anti-IFNγ antibody blocked M1 polarization induced by rIL9 treatment as much as the addition of anti-IL9 (Fig. 1J). In contrast, the neutralization of IFNγ did not affect macrophage proliferation stimulated by rIL9 (Fig. 1K). From these results, we conclude that IL9 induced M1 polarization in an IFNγ-dependent manner. However, the efficacy of IL9 on macrophage proliferation appeared to be independent of IFNγ.

### IL9 Repolarizes M2-like Macrophages Toward the M1-like Phenotype

**FIGURE 2:**
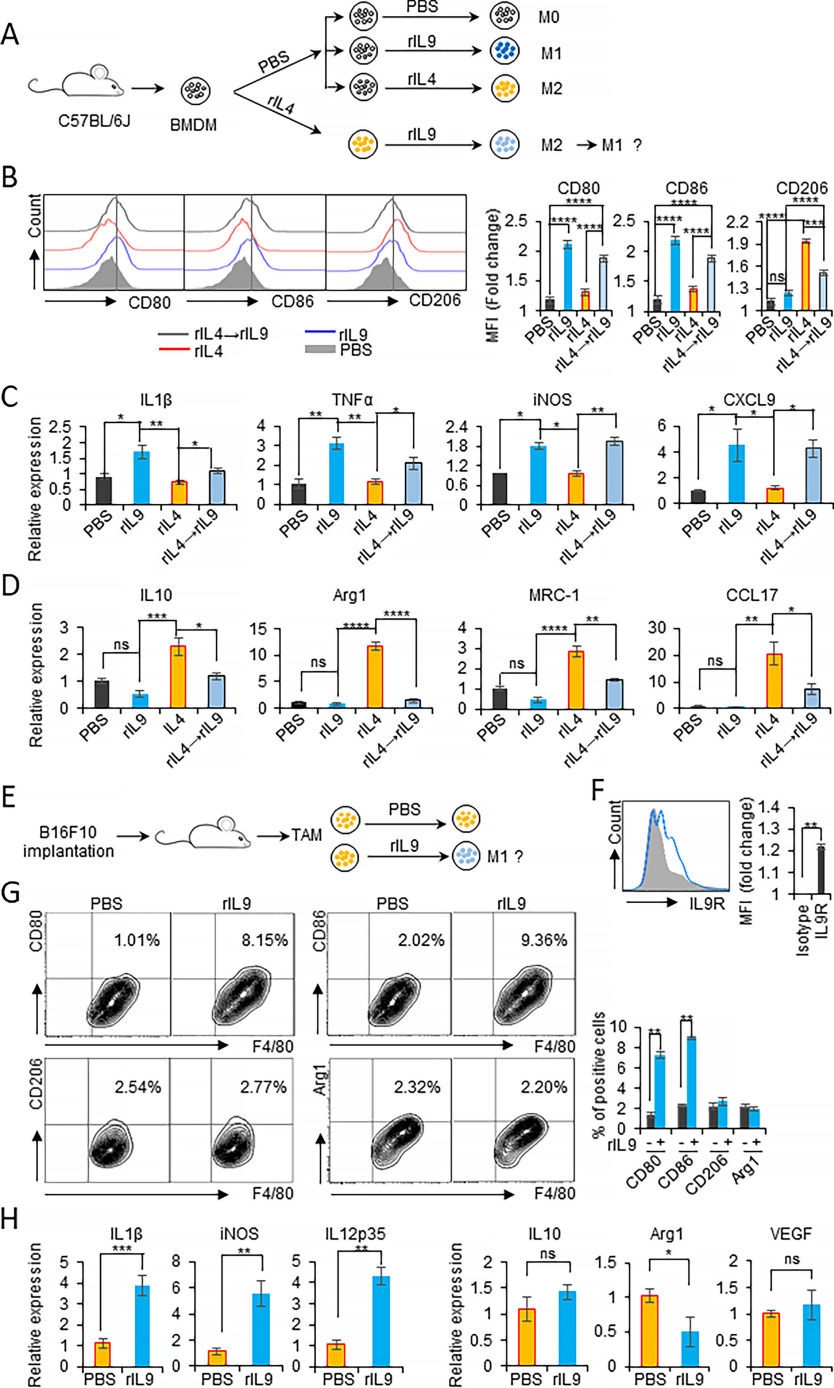
IL9 treatment repolarizes M2 macrophages and TAMs to the M1-like phenotype. **A–D,** BMDMs were treated with 20 ng/mL rIL4 for 24 hours. After washing off the rIL4, the IL4-treated BMDMs were retreated with 20 ng/mL rIL9 for 48 hours. Groups of BMDMs were treated with either 20 ng/mL rIL9 or 20 ng/mL rIL4 as the positive and negative controls, respectively (**A**). FACS analysis of M1/M2 markers on the surface of the treated cells by staining with anti-CD80, anti-CD86, and anti-CD206. Bar graphs of the histogram analysis are shown (right; **B**). qPCR analysis of the mRNA expression of M1 markers (**C**) or M2 markers (**D**) in the treated groups as indicated. **E–H,** A total of 4 × 10^5^ B16F10 cells were subcutaneously implanted into mice. Two weeks after the implantation, the tumor masses were collected, and TAMs were sorted using a TAM isolation kit (**E**). Expression of IL9R on isolated TAMs is examined by FACS (**F**). The isolated TAMs were treated with 100 ng rIL9 or PBS for 48 hours. The expression of M1 (CD80, CD86) and M2 (CD206 and Arg1) markers was analyzed by FACS (**G**) and the relative mRNA expression of other M1 markers (IL1β, iNOS, IL12p35) and M2 markers (IL10, Arg 1, VEGF) was analyzed by qPCR (**H**). *, *P* < 0.05; **, *P* < 0.01; ***, *P* < 0.001; and ****, *P* < 0.0001.

To investigate whether IL9 treatment could repolarize macrophages from the M2- to the M1-like phenotype, rIL4-polarized BMDMs were treated with rIL9 as described in Fig. 2A. Treatment of rIL4-pretreated BMDMs with rIL9 induced CD80 and CD86 expression by 1.4-fold and reduced the expression of CD206, an M2 marker, by 1.3-fold compared with rIL4 treatment alone, implying that treatment with IL9 might be efficient in reverting macrophages from the M2 to the M1 phenotype (Fig 2B). We further analyzed the gene expression of other M1 and M2 markers (Fig. 2C and D). rIL9 treatment of IL4-pretreated BMDMs significantly provoked the gene expression of M1-associated genes (Fig. 2C) and dramatically lowered M2-related genes (Fig. 2D) compared with IL4 treatment only. To address the role of IL9 signaling on the polarization of TAMs *in vitro*, we subcutaneously implanted B16F10 melanoma into C57BL/6J mice. Fifteen days after tumor implantation, TAMs from the tumor masses were sorted (Fig. 2E). The high expression of IL9R in sorted TAMs was shown by FACS analysis (Fig. 2F). When TAMs were treated with rIL9, the percentage of CD80^+^F4/80^+^ and CD86^+^F4/80^+^ M1 cells was significantly enhanced, but CD206^+^F4/80^+^ and Arg1^+^F4/80^+^ M2 cells were not changed, indicating that IL9 induced the M1-like polarization of TAMs (Fig. 2G). The mRNA analysis was also used to verify the M1 polarization of TAMs by IL9. IL1β, iNOS, and IL12p35 expression levels were significantly induced, but IL10, Arg1 and VEGF levels were either unchanged or reduced (Fig. 2H). These data collectively indicate that IL9 repolarized IL4-induced M2 macrophages and melanoma-derived TAMs toward the M1-like phenotype.

### The Combination of IL9 and Macrophages Delays the Growth of B16F10 Melanoma in Mice

**FIGURE 3:**
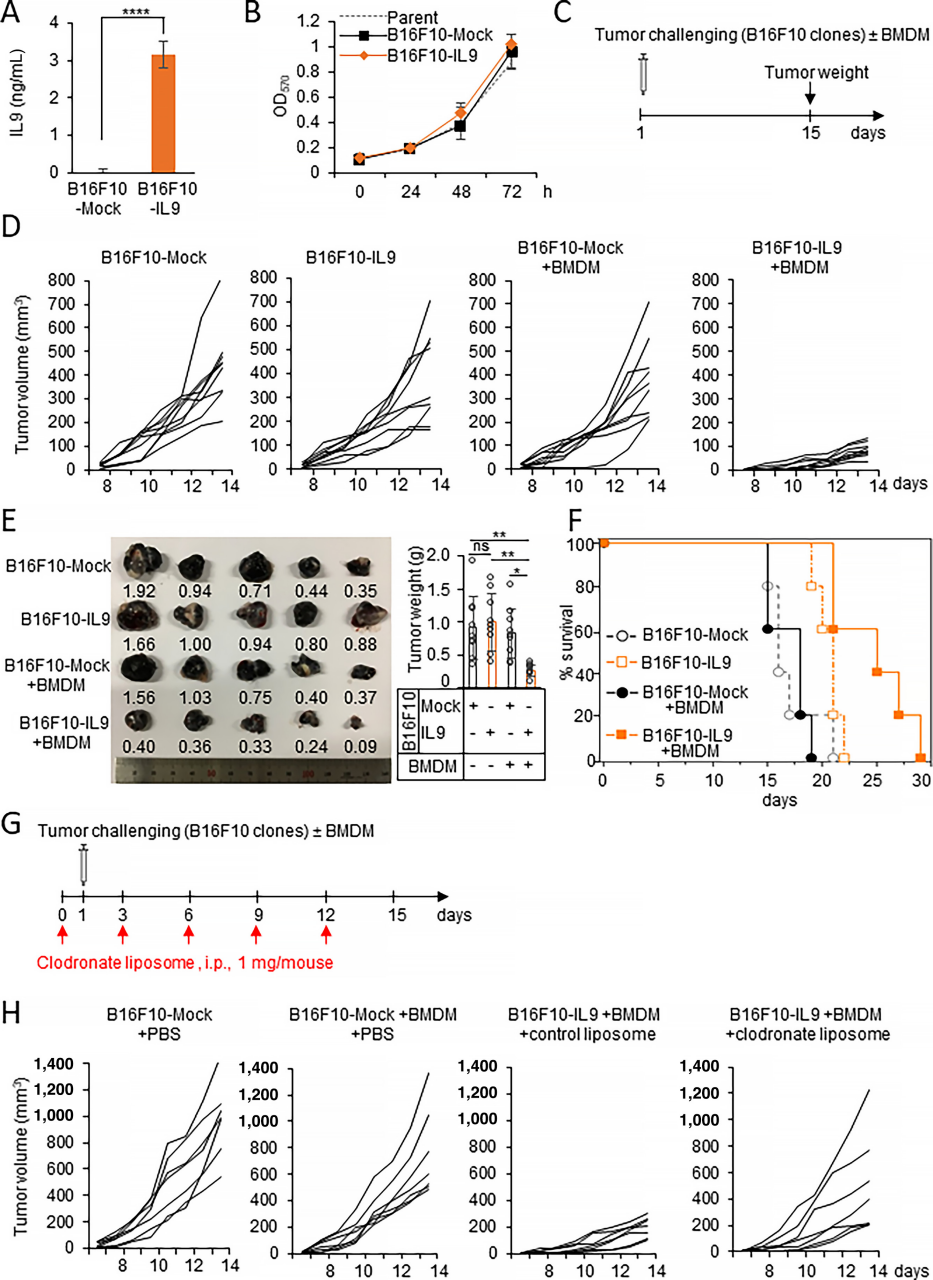
Coimplantation of macrophages with IL9-secreting B16F10 cells delays the growth of the tumors in mice. **A,** IL9 secreted from B16F10-IL9 stable clones was measured by ELISA. The mock vector–transfected B16F10-Mock clone was used as the control. **B,** MTT assays were performed to compare the proliferation of B16F10-IL9, B16F10-Mock, and B16F10-parent cells. **C–F,** Mice (*n* = 9) were subcutaneously injected with a mixture of 4 × 10^5^ B16F10 cells (B16F10-IL9 or B16F10-Mock) and 2 × 10^5^ BMDMs. Groups of mice (*n* = 9) were injected with 4 × 10^5^ B16F10-IL9 or B16F10-Mock as controls (**C**). The tumor growth (**D**) of the tumor-bearing mice is shown. On day 15 after tumor inoculation, the mice were euthanized. The picture (left) and the weight of the tumor masses (right) were recorded and shown (**E**). The survival of tumor-bearing mice is shown in **F**. **G** and **H,** Mice (*n* = 7) were treated with either clodronate liposomes, control liposomes, or PBS at 3-day intervals, as indicated by the red arrows. One day after the first clodronate treatment, the mice were injected with B16F10-Mock, B16F10-Mock+BMDM, or B16F10-IL9+BMDM (**G**). Tumor growth was measured daily (**H**). *, *P* < 0.05; **, *P* < 0.01; and ****, *P* < 0.0001.

To further study the roles of IL9 in the TAM population, we adopted the coinjection system of B16F10 melanoma cells plus macrophages as described in previous studies (17–19). To generate a consistent expression of IL9 in the tumor microenvironment (TME), we used a stable B16F10 cell line secreting IL9, called B16F10-IL9. B16F10-Mock was the control cell line. One million B16F10-IL9 cells secreted IL9 into the culture medium at concentrations as high as 3 ng/mL for 24 hours, while the levels secreted by the B16F10-Mock cells were not detectable (Fig. 3A). Because the B16F10 melanoma cells did not express IL9R on their surface (Supplementary Fig. S4), autocrine IL9 expression did not affect the *in vitro* proliferation of IL9-B16F10 cells (Fig. 3B). The transfected B16F10 cells were implanted subcutaneously into the right flank of C57BL/6J mice (*n* = 9) along with BMDMs (Fig. 3C). To allow the macrophages to enter the tumor mass, BMDMs and tumor cells were mixed at a 1:2 ratio and then embedded in Matrigel to allow them to solidify immediately upon injection. B16F10-Mock–injected mice showed similar tumor volume growth both in the presence and absence of BMDMs. No statistical difference in the *in vivo* growth between B16F10-Mock and B16F10-IL9 cells was observed. However, mice implanted with B16F10-IL9+BMDMs showed dramatically reduced tumor growth compared with mice injected with B16F10-Mock+BMDMs or with B16F10-IL9 alone (Fig. 3D). On day 15 after tumor inoculation, the mice were euthanized, and tumor masses were collected (Fig. 3E). The tumor weight in the mice in the B16F10-IL9+BMDM group was 2.8-fold lower than that of the mice in the B16F10-Mock+BMDM group. The survival of the mice in the B16F10-IL9+BMDM group was extended for 10 days compared with that of the B16F10-Mock or B16F10-Mock+BMDM groups (Fig. 3F). No significant change in the bodyweight of the tumor-bearing mice was observed between the groups for 13 days after tumor implantation (Supplementary Fig. S5). The results suggest that the enrichment of TAMs and IL9 in the TME evoked effective antitumor activity against B16F10 melanoma. To further confirm the TAM contribution, we tried to deplete TAMs using clodronate liposomes (13, 20). First, the selective cytotoxicity of clodronate liposomes on BMDMs was tested *in vitro* (Supplementary Fig. S6). Treatment with clodronate liposomes selectively killed BMDMs, but not B16F10 cells, in a concentration-dependent manner (IC_50_ = 0.415 mg/mL). As illustrated in Fig. 3G, mice (*n* = 7) were intraperitoneally treated with clodronate liposomes one day before the transfected B16F10 cells were implanted. The treatment was repeated every 3 days until day 12 after tumor implantation. The efficacy of clodronate liposome treatment was validated on day 12 after tumor implantation. Intraperitoneal injection with clodronate liposomes significantly depleted TAMs *in vivo* (Supplementary Fig. S6B). In B16F10-IL9+BMDM mice treated with clodronate liposomes, tumors grew in 4 of 7 mice and reached a tumor volume similar to that of the B16F10-Mock and B16F10-Mock+BMDM groups, whereas the control liposome treatment failed to prevent the reduction in tumor volume (Fig. 3H). These data imply that macrophage-scavenging diminished the antitumor responses of IL9 in mice injected with B16F10-IL9+BMDMs. Collectively, our data suggest that intratumoral IL9 induced antitumor responses in a macrophage-dependent manner.

### The Combination of IL9 and Macrophages Induces the Accumulation of Immune Cells in the Tumor

**FIGURE 4:**
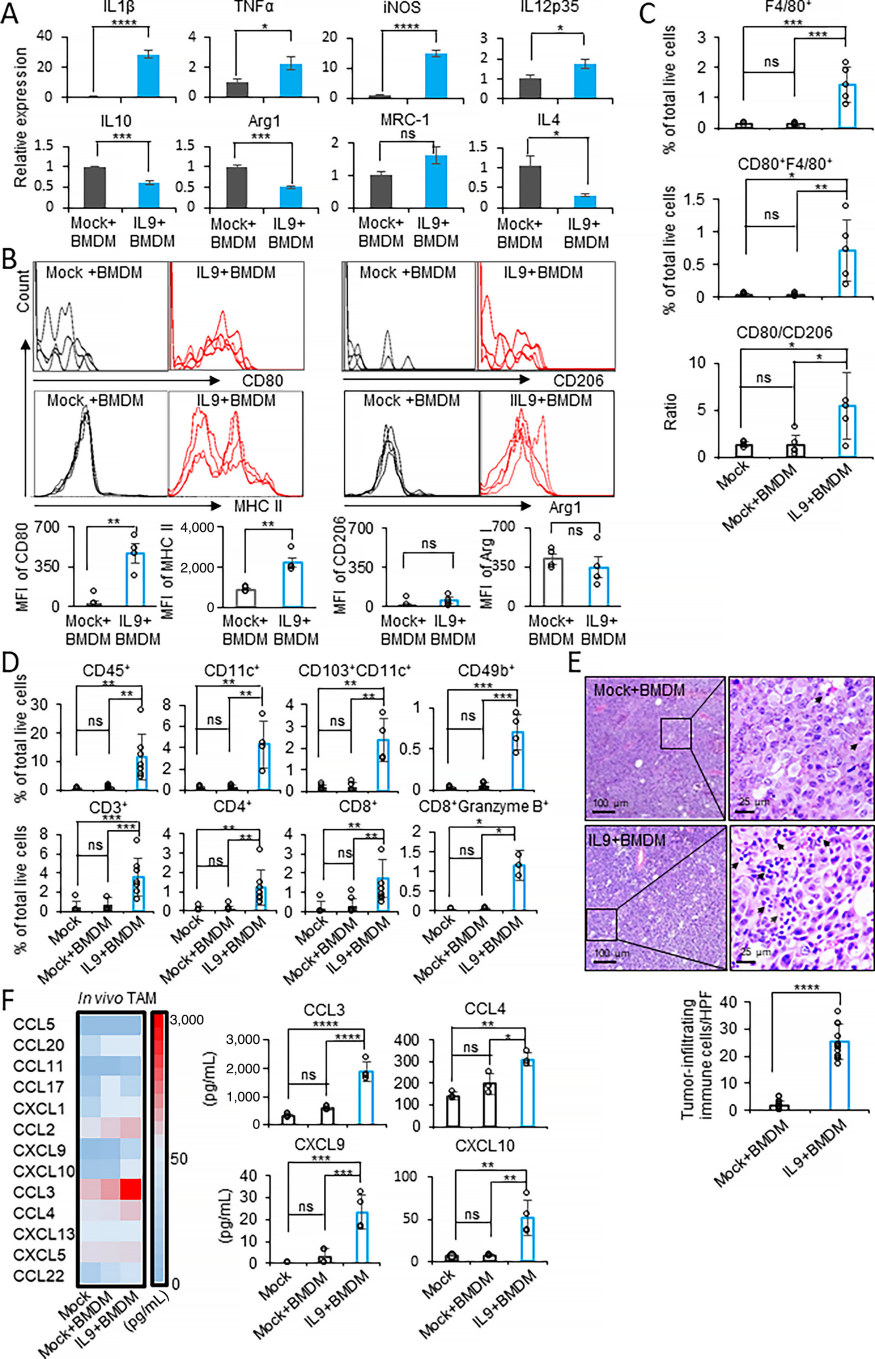
Intratumoral IL9 directly polarizes TAMs to the M1-like phenotype and recruits antitumor immune cells into the TME. **A–D,** Mice (*n* = 4) were challenged as described in Fig. 3C and euthanized on day 15 after tumor implantation. The tumor masses were collected, and TAMs were sorted using a TAM isolation kit. **A,** qPCR analysis of the mRNA expression of M1/M2 markers in TAMs isolated from the B16F10-IL9+BMDM (IL9+BMDM) and B16F10-Mock+BMDM (Mock+BMDM) groups. **B,** FACS analysis of CD80, MHC class II, CD206, and Arg1 on TAMs (CD45^+^CD11b^+^F4/80^+^). Bar graphs of the MFI in the histograms are shown (bottom). **C,** The percentage of F4/80^+^(CD45^+^CD11b^+^F4/80^+^), CD80^+^F4/80^+^ (CD45^+^CD11b^+^F4/80^+^CD80^+^) cells and the ratios of CD80^+^F4/80^+^/CD206^+^F4/80^+^ in each group are shown. **D,** The percentages of tumor-infiltrating immune cells (CD45^+^), DCs (CD11c^+^), CD103^+^ DCs (CD103^+^CD11c^+^), NK cells (CD49b^+^), T cells (CD3^+^CD4^+^ or CD3^+^CD8^+^), and cytotoxic effector T cells (CD3^+^CD8^+^granzyme B^+^) were stained and analyzed by FACS. **E,** Mice (*n* = 4) were challenged as described in Fig. 3. Mice injected with B16F10-IL9+BMDM or B16F10-Mock+BMDM were euthanized on day 11. Tumor mass sections were stained with H&E. The arrows identify immune cells infiltrating the TME. The left and right sections are magnified 50× and 200×, respectively. The number of tumor-infiltrating immune cells per high power field (HPF) is shown in the bar graph. **F,** Mice (*n* = 4) were challenged as described in Fig. 3C and euthanized on day 11 after tumor implantation, and the tumor-infiltrating macrophages were isolated using a TAM isolation kit. The chemokine expression in the cell lysate from the TAM fraction was analyzed by multiplex bead array. A heatmap of chemokine profiles (left) and the individual CCL3, CCL4, CXCL9, and CXCL10 concentrations (right) in the cell lysate of TAMs isolated from each group are shown. *, *P* < 0.05; **, *P* < 0.01; ***, *P* < 0.001; and ****, *P* < 0.0001.

To investigate the mechanism underlying the antitumor effect of IL9 on the macrophage-enriched tumor model, we collected tumor masses after euthanizing the tumor-bearing mice (*n* = 4) on day 15 after tumor implantation (Fig. 3C). F4/80^+^ TAMs from the tumor masses were sorted for further analysis. As shown in Fig. 4A, TAMs from B16F10-IL9+BMDM mice had a higher expression of M1 markers and lower expression of M2 markers, except for MRC-1, compared with mice in the B16F10-Mock+BMDM group. TAMs from growing tumors were also characterized by surface or intracellular staining with anti-CD80, anti-MHC class II, anti-CD206, and anti-Arg1 antibodies (Fig. 4B). The FACS gating used for this analysis is shown in Supplementary Fig. S7. Consistent with the gene expression data, the surface expression of CD80 and MHC class II on TAMs from the B16F10-IL9+BMDM group was 19-fold and 2.6-fold higher, respectively, than in the B16F10-Mock+BMDM group. No statistical difference in the expression of CD206 and Arg1 was found between these groups. Of note, the absence of CD80^+^ macrophages in the tumors was related to the immune-suppressive response of TAMs and short overall survival (25), and the repolarization of TAMs to M1 could effectively provoke an antitumor response (2, 26). Hence, TAM subsets were further validated by comparing the percentage of TAMs and CD80^+^ TAMs among the groups (Fig. 4C). The percentage of TAMs was increased 12-fold in the B16F10-IL9+BMDM group. Particularly, the percentage of M1-like TAMs (CD80^+^F4/80^+^) was 27-fold higher in the B16F10-IL9+BMDM group. Moreover, the M1/M2 ratio (CD80^+^ TAM/CD206^+^ TAM) was 4.4-fold higher in TAMs isolated from the B16F10-IL9+BMDM group. This ratio suggested a tendency toward expressing M1 functional molecules in the TAMs from B16F10-IL9+BMDM–injected mice. Altogether, the data demonstrated that intratumoral supplementation with IL9 polarized TAMs to the M1-like phenotype.

Next, we investigated changes in tumor-infiltrating lymphocyte (TIL) subsets that contributed to antitumor immunity in the same TAM samples (Fig. 4D). The FACS gating used in TIL analysis is shown in Supplementary Fig. S7. The percentage of tumor-infiltrated CD45^+^ immune cells in the B16F10-IL9+BMDM group was increased 15-fold compared with the B16F10-Mock+BMDM group. Among the immune subsets, the percentage of tumor-infiltrated NKs (CD49b^+^), DCs (CD11c^+^), and T cells (CD3^+^) in the B16F10-IL9+BMDM group increased by 15-, 13-, and 5.5-fold, respectively. Further analysis revealed a 12-fold increase in the proportion of tumor-infiltrated CD103^+^ DCs in the B16F10-IL9+BMDM group, which is a crucial driver of intratumoral CD8^+^ T-cell activation and antitumor immunity induction (27). Among the T-cell subpopulations, the percentage of both tumor-infiltrated CD4^+^ T cells and CD8^+^ T cells was increased by 10- and 6-fold, respectively, in the tumors of the B16F10-IL9+BMDM group. Notably, the infiltration of effector CD8^+^ T cells with cytotoxic capability (CD8^+^granzyme B^+^ T cells) was significantly higher (22-fold) in the tumors of the B16F10-IL9+BMDM group. The microscopic analysis of H&E stains of tumor tissues provided evidence of immune cell infiltration into tumor tissues in the presence of IL9. Although only a few immune cells were noted in the tumor sections of the B16F10-Mock+BMDM group, the accumulation of infiltrating immune cells was prominent in the B16F10-IL9+BMDM group sections, which was 14-fold higher than that in the B16F10-Mock+BMDM group (Fig. 4E). Together, these data suggest that the combination of intratumoral IL9 and TAMs induced a potent antitumor immune response by promoting the accumulation and activation of DCs, NK cells, and cytotoxic T cells in the TME. Given the possibility that intratumoral IL9 is associated with the accumulation of antitumor immune cells in the tumor mass, we sought to determine which chemotactic chemokines could play an important role in our experimental model. The cell lysate of TAM fractions isolated from B16F10-IL9+BMDM and B16F10-Mock+BMDM–injected mice were examined using a multiplex bead array to analyze the expression profiles of mouse chemotactic chemokines. As shown in Fig. 4F, the expression of four of 13 proinflammatory factors was significantly elevated in the TAMs of the B16F10-IL9+BMDM group, including CCL3 (1,880 pg/mL, 3-fold increase), CCL4 (309 pg/mL, 1.5-fold increase), CXCL9 (23.32 pg/mL, 8-fold increase), and CXCL10 (51.8 pg/mL, 7-fold increase). Taken together, the combination of IL9 and macrophages induced the accumulation of immune cells in the tumor, probably by producing chemotactic chemokines.

### IL9-polarized Macrophages Induce the Expression of Chemokines, Resulting in the Chemotactic Recruitment of Antitumor Immune Cells

**FIGURE 5:**
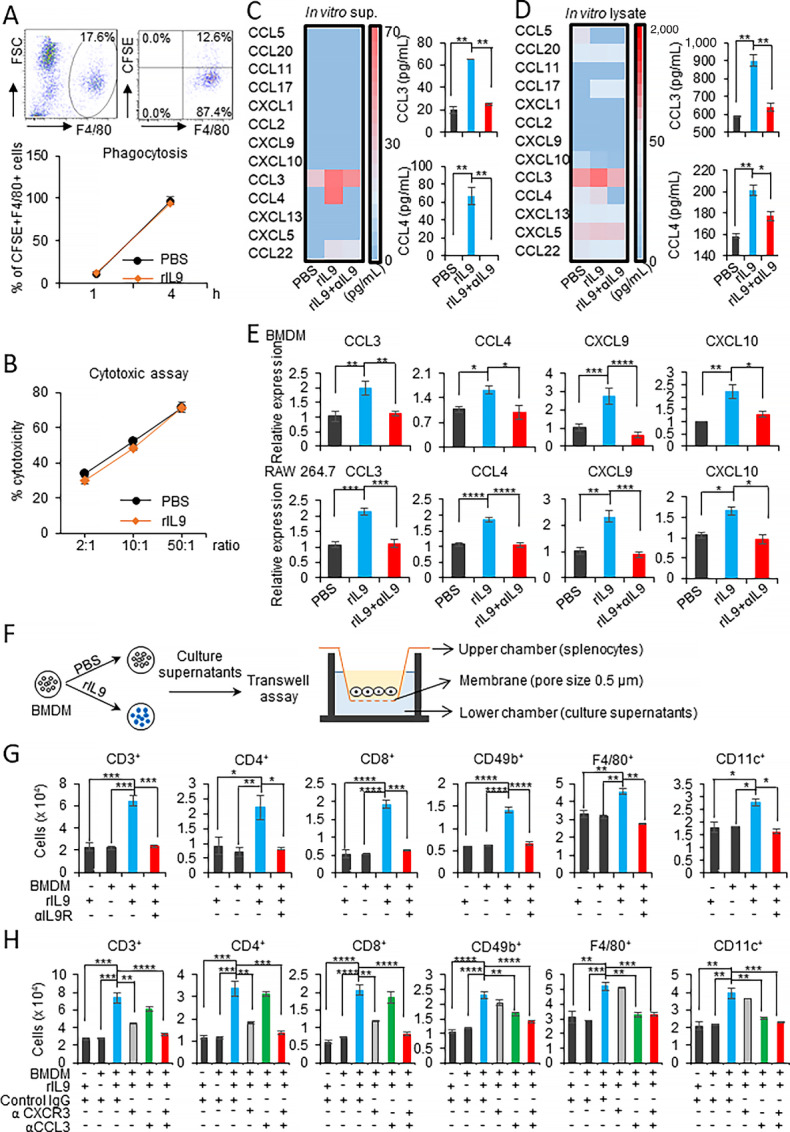
IL9-polarized macrophages induce chemokine secretion and the chemotactic recruitment of immune cells. **A,** The phagocytic activity of BMDMs treated with 100 ng rIL9 was evaluated against CFSE-labeled apoptotic B16F10 cells. At the indicated timepoints, the cells were harvested and the percentage of the F4/80^+^CFSE^+^ population was analyzed. **B,** IL9-treated BMDMs and B16F10 target cells were cocultured at the indicated ratios. The viability of the cocultured cells was analyzed after 48 hours using the MTT assay. The percentage of cytotoxicity was calculated as described in the Materials and Methods. **C** and **D,** BMDMs were treated with 100 ng/mL rIL9, the mixture of rIL9+2.5 μg/mL anti-IL9R antibody, or PBS for 48 hours, and chemokine profiles were determined by multiplex bead array. The heatmaps and CCL3 and CCL4 concentrations in the culture supernatants (**C**) and cell lysates obtained from the samples treated with 3 μmol/L momensine for 6 hours (**D**) are shown. **E,** qPCR analysis of the mRNA expression of chemokines in BMDMs and RAW264.7 macrophages treated with 100 ng/mL rIL9, rIL9+2.5 μg/mL anti-IL9R, or PBS for 48 and 24 hours, respectively. **F–H,** Schematic image illustrates the transwell assay evaluating the ability of rIL9-treated BMDMs to induce the chemotactic migration of immune cell subsets (**F**). BMDMs were treated with rIL9 or the mixture of rIL9+1 μg/mL anti-IL9R for 48 hours. The culture supernatants were collected and placed in the lower transwell chambers. Fresh non-cultured medium containing 100 ng/mL rIL9 was used as the negative control. Splenocytes from C57BL/6J mice were isolated and placed in the upper chambers. After 3 hours of incubation, the migrated cell subsets were harvested, and the cell numbers were determined by FACS analysis (**G**). BMDMs were treated with 100 ng rIL9 for 48 hours. To determine whether cell migration was affected by blocking CCL3, the culture supernatants were treated with 1.5 μg/mL anti-CCL3 or control IgG antibodies and then loaded into the lower transwell chamber. Splenocytes from C57BL/6J mice were placed in the upper chambers. To examine the role of CXCL9/10 in the migration, their receptor, CXCR3, was blocked. Splenocytes were pretreated with 3.8 μg/mL anti-CXCR3 or isotype control and then loaded into the upper chamber (**H**). *, *P* < 0.05; **, *P* < 0.01; ***, *P* < 0.001; and ****, *P* < 0.0001.

To clarify the effect of IL9 treatment on macrophage functions, we conducted macrophage functional assays *in vitro*. Firstly, we determined whether IL9 treatment increased the phagocytosis of BMDMs. CFSE-labeled apoptotic B16F10 cells were used as target cells for opsonin-independent phagocytosis. As shown in Fig. 5A, the percentage of CFSE^+^F4/80^+^ macrophages was similar between rIL9-treated and PBS-treated BMDMs for 1 to 4 hours, so no significant difference in phagocytosis among the groups was observed. Second, to compare the cytotoxic effect of IL9-polarized macrophages, rIL9 and PBS-treated BMDMs were used as effector cells while B16F10 tumor cells were used as the targets. The cytotoxicity of rIL9 or PBS-pretreated BMDMs was measured after 48 hours of incubation with three ratios (2:1, 10:1, and 50:1) of target cells. As presented in Fig. 5B, there was no significant difference in cytotoxicity against B16F10 cancer cells between IL9-polarized BMDMs and PBS-treated controls.

As shown in Fig. 4, the *in vivo* data revealed that intratumoral IL9-polarized TAMs could play a central role in recruiting antitumor immune cells and facilitating their infiltration into the tumor. The migration and infiltration of proper immune cells into a solid tumor are crucial for successful antitumor responses (28). Therefore, we sought to identify the critical proinflammatory chemokines originating from IL9-polarized macrophages. BMDMs were treated with PBS, rIL9, or a mixture of rIL9+anti-IL9 antibody. Then, the culture supernatants and monensin-treated cell lysates were tested to evaluate the expression of 13 proinflammatory chemokines using a multiplex bead array. Consistent with the data using TAMs in Fig. 4, the expression of CCL3 and CCL4 was elevated in both culture supernatants and cell lysates of the rIL9-treated group. This expression was abolished by neutralizing IL9 (Fig. 5C and D). The levels of secreted CCL3 and CCL4 chemokines detected in the culture supernatant from rIL9-treated BMDMs were about 65 pg/mL. The level of CCL3 and CCL4 in the cell lysates from rIL9-treated BMDMs was 1.6-fold (900 pg/mL) and 1.3-fold (200 pg/mL) higher, respectively, than those from the PBS-treated group. Compared with the chemokine expression profile of TAMs, no expression of CXCL9 and CXCL10 in rIL9-treated BMDMs was detected by the multiplex assay. This finding could be due to their low secretion levels. Therefore, the gene expression levels of the potent chemotactic chemokines were quantitated by qPCR (Fig. 5E). In the BMDMs, the relative expression of CCL3, CCL4, CXCL9, and CXCL10 in the rIL9-treated group was increased 2.0-, 1.5-, 2.6-, and 2.2-fold, respectively, compared with the PBS-treated group. Similar to the BMDMs, the relative expression of CCL3, CCL4, CXCL9, and CXCL10 in rIL9-treated RAW 264.7 cells was increased 2.0-, 1.7-, 2.3-, and 1.6-fold, respectively, compared with the PBS-treated group. The addition of anti-IL9 antibody abolished these stimulation effects of rIL9 on chemokine secretion by macrophages.

To evaluate the chemotaxis of immune cells that occurred in response to chemoattractants derived from IL9-polarized macrophages, we performed the transwell assay to assess the chemotactic effect of culture supernatant from rIL9-treated BMDMs on total splenocytes (Fig. 5F). The migrating cells were collected and labeled for FACS analysis of the immune cell subsets as shown in Supplementary Fig. S8. As shown in Fig. 5G, T cells, NK cells, macrophages, and DCs responded to the culture supernatant from rIL9-treated BMDMs and showed enhanced migration. The increased migration of all of the immune cells was completely inhibited when neutralizing anti-IL9R antibody was added to rIL9-treated BMDMs. CCL3 and CCL4 are the two major forms of macrophage inflammatory protein 1 (MIP-1). Because they have high structural similarity and largely redundant functions, CCL3 was chosen for further analysis. The culture supernatant from rIL9-treated BMDMs was collected and pretreated with neutralizing anti-CCL3 or control IgG antibody before the transwell assay. The inhibition of CCL3 showed a strong inhibitory effect on the migration of macrophages, DCs, and NK cells, but only weakly blocked the chemotaxis of CD4^+^ or CD8^+^ T cells (Fig. 5H). To determine whether blocking CXCL9 and CXCL10, ligands of the CXCR3 receptor, influenced the chemotaxis of immune cell subsets, we pretreated splenocytes with either anti-CXCR3 or control antibodies for chemotaxis inhibition assays. As expected, blocking CXCR3 using the anti-CXCR3 antibody significantly inhibited T-cell and NK-cell chemotaxis induced by the culture supernatant of IL9-polarized BMDMs, whereas blocking CXCR3 had a moderate effect on DC migration and almost no effect on the migration of macrophages (Fig. 5H). Notably, the combination of anti-CXCR3 and anti-CCL3 antibodies completely inhibited the chemotaxis of splenic CD4^+^/CD8^+^ T cells, NK cells, macrophages, and DCs induced by IL9 treatment, suggesting that IL9-polarized BMDMs stimulated immune cell chemotaxis in MIP-1 and CXCR3 ligand–mediated manners.

### Recombinant IL9 Treatment Delays the Growth of Cancer Coimplanted with Macrophages

**FIGURE 6:**
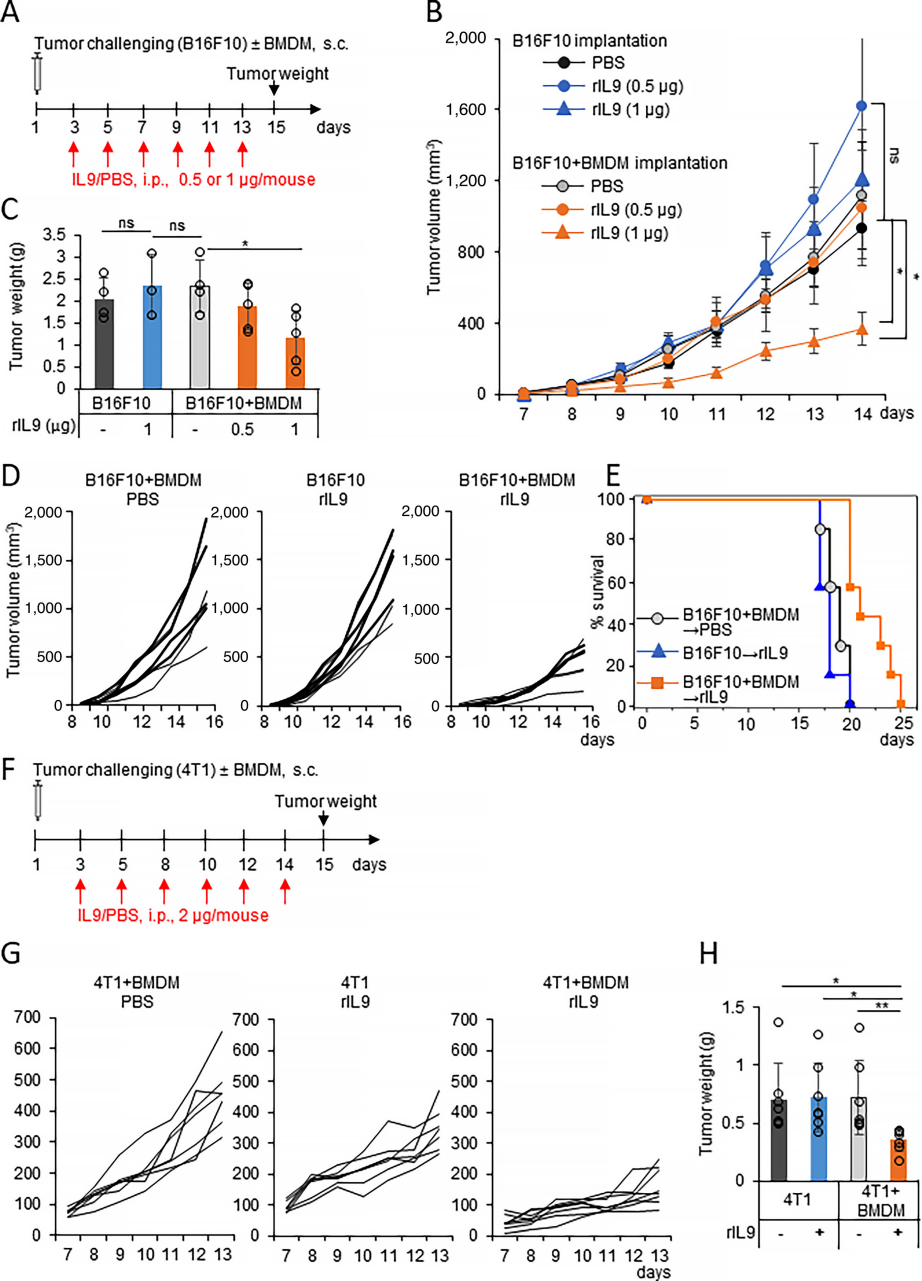
IL9 delays the growth of cancer cells implanted with macrophages in mice. **A–C,** Mice (*n* = 4–6) were injected with either 4 × 10^5^ B16F10 or the mixture of 4 × 10^5^ B16F10 + 2 × 10^5^ BMDMs. The tumor-bearing mice were treated with either IL9 (0.5 or 1 μg/mouse) or PBS every 2 days for 2 weeks, as indicated by the red arrows (**A**). Tumor growth (**B**) and tumor weight at day 15 (**C**) are shown. **D** and **E,** Mice (*n* = 7) were injected with either 2 × 10^5^ B16F10 or 2 × 10^5^ B16F10 + 1 × 10^5^ BMDMs. The tumor-bearing mice were treated with either IL9 (1 μg/mouse) or PBS at 2-day intervals for 2 weeks. Tumor growth (**D**) and survival (**E**) of the mice are shown. **F** and **H,** Mice (*n* = 7) were injected with either 4 × 10^5^ 4T1 or the mixture of 4 × 10^5^ 4T1 + 2 × 10^5^ BMDMs and treated with either IL9 (2 μg/mouse) or PBS every 2 days, as indicated by the red arrows (**F**). Tumor growth (**G**) and tumor weight of the mice on day 14 (**H**) are shown. *, *P* < 0.05 and **, *P* < 0.01.

Next, rIL9 was used to evaluate the therapeutic effect of the systemic circulation of IL9. Mice were treated intraperitoneally with rIL9 at different doses at 2-day intervals after the subcutaneous implantation of B16F10 cells along with BMDMs (Fig. 6A). An rIL9-treated group injected with B16F10 cells only and PBS-treated groups after the implantation of B16F10 cells only or B16F10+BMDMs were included as controls. rIL9 treatment significantly delayed tumor growth in the B16F10+BMDM groups in a dose-dependent manner, but not in the B16F10-only inoculated group (Fig. 6B). No significant difference was found between the PBS-treated groups after B16F10-only and B16F10+BMDM implantation. Tumor weights on day 15 in the rIL9-treated group implanted with B16F10+BMDMs were lower than those in the controls groups by 1.8-fold at a dosage of 1 μg/mouse (Fig. 6C). No significant difference in body weight of the mice was observed between all treated groups (Supplementary Fig. S9A). In the experiment using more mice at an rIL9 dose of 1 μg/mouse, the growth of tumors in the rIL9-treated B16F10+BMDM group was significantly inhibited (Fig. 6D), and the survival of those mice was also extended by around 5 days compared with the rIL9-treated B16F10 group (Fig. 6E). Because 4T1 breast cancer cells did not express IL9R on their surface (Supplementary Fig. S4), the therapeutic effect of IL9 therapy on macrophage-enriched 4T1 breast cancer was also evaluated (Fig. 6F–H). After subcutaneous implantation with either 4T1 breast cancer cells or a mixture of 4T1+BMDMs, BALB/C mice were treated intraperitoneally with rIL9 or PBS at 2-day intervals (Fig. 6F). As in the melanoma case, rIL9 treatment delayed tumor growth after implantation in the 4T1+BMDM groups compared with the 4T1-only implantation group (Fig. 6G). Compared with the controls, tumor weights on day 15 in the rIL9-treated 4T1+BMDM group were lower by 2-fold (Fig. 6H). No significant difference in body weights was observed between the experimental groups (Supplementary Fig. S9B). Collectively, these results demonstrated that recombinant IL9 treatment delayed the growth of cancers and enhanced survival when cancer cells were implanted with macrophages. Therefore, the results indicated that the anticancerous role of macrophages could be evoked when it was signaled and activated by IL9.

## Discussion

Recently, strategies to reeducate TAMs have attracted increased scientific attention due to the potential benefits of combining them with immune checkpoint inhibitors (CPI; refs. 29, 30). In this research, we found that IL9 had a strong potential to reeducate macrophages, including TAMs, to stimulate the antitumor immune response in an IFNγ-dependent manner. IL9-IL9R signaling polarized macrophages to the M1 phenotype and also promoted their proliferation. IL9 retrains M2 macrophages or TAMs to the M1 phenotype. We specifically showed that IL9-polarized TAMs induced antitumor immunity in mice by recruiting antitumor immune cells into the tumor mass via releasing the macrophage-derived chemoattractants, CCL3/4 and CXCL9/10. In addition, when macrophages were enriched, intraperitoneal and intratumoral treatment with rIL9 delayed the growth of B16F10 melanoma and 4T1 breast cancer cells.

Here, we observed that the surface expression of IL9R was common on macrophage cell lines and primary macrophages isolated from mice. In addition, IL9 treatment stimulated proliferation and induced STAT1 and STAT3 phosphorylation in macrophages. Our observations are consistent with previous reports on the growth-promoting effect of IL9 in other hematopoietic cells such as T cells, B cells, and NK cells (7). Regarding the effect of IL9 on macrophages, M1/M2 polarization has not been extensively studied. In B16F10 lung metastasis and allergic lung inflammation models, lung macrophages responded to IL9 stimulation to promote proinflammatory immune responses (12, 31). In contrast, in the multiple sclerosis brain system, IL9 acted on macrophages to enhance the production of TGFβ, consequently inducing an anti-inflammatory response (32). In this study, we showed that *in vitro* stimulation with IL9 polarized macrophages to the proinflammatory M1 phenotype, and IL9 treatment converted M2 plastic BMDMs to the M1 phenotype by dampening M2-associated markers in IL4-treated M2 macrophages. This effect on M1 polarization was found through the autocrine effect of IFNγ secreted from IL9-stimulated macrophages. These IL9 properties, combined with the IL9 activation of macrophage proliferation, are important for the proinflammatory role of TAMs. Because of the reversibility of the macrophage M1/M2 activation state, the newly established microenvironment could influence the TAM reprogramming process. Therefore, retraining M2 macrophages to the M1 phenotype has been proposed as the ideal therapeutic strategy in cancer treatment.

Macrophages represent approximately 5% to 7% of the total lung cells and about 80% of lung immune cells (33). For lung cancer research, although the protumor function of macrophages has been reported, accumulating evidence supports the antitumoral functions of macrophages (34–36). For example, increases in tumor islet CD68^+^ macrophage density and the tumor islet and/or stromal macrophage ratio were significant independent predictors of increased survival in patients with surgically resected non–small cell lung cancer (35). Our previous study found that the number of tumor nodules in the lungs of the mice intravenously injected with IL9-expressing B16F10 was significantly less than that of the control groups, and pulmonary M1 macrophages were the population most significantly increased in the lungs of those mice (12). Therefore, we prespecified that intratumoral supplementation with IL9 might alter the TME of TAM-enriched tumors and convert their immunosuppressive phenotype. For this purpose, an IL9-secreting tumor cell line that evenly and continuously distribute IL9 throughout the tumor mass, was used for syngeneic implantation. A coinjection system of BMDMs and tumor cells was applied to mimic TAMs in the TME. The mixed injection of tumor cells and BMDMs was verified in previous studies as a useful tool to investigate the antitumor effect of M1-polarized TAMs (17–19). In our experiment, we characterized BMDMs and TAMs sorted from B16F10 tumor masses. Both TAMs and BMDMs expressed IL9R on the cell surface. Treatment with IL9 drove both macrophages to the M1 phenotype because IL9 similarly induced the mRNA and protein expression of M1 markers and did not significantly alter M2 markers. Therefore, we used BMDMs as a proper macrophage source to mimic the TAM-enriched microenvironment in an *in vivo* mice model. Using the coinjection model, we found that the intratumoral combination of IL9 and TAMs delayed the growth of B16F10 melanoma in mice. At first, the presence of IL9 within the tumor area was essential to generating proper antitumor immunity. Even though just a few nanograms of IL9 were secreted by the B16F10-IL9 mice per day, the consistent and even distribution of IL9 in the tumor area resulted in a strong antitumor response. Notably, without macrophage supplementation, there was no significant difference between the B16F10-Mock and B16F10-IL9 groups, and the depletion of TAMs after tumor cell and BMDM coimplantation by treatment with clodronate liposomes abolished the antitumor responses in B16F10-IL9+BMDMs. Our data presented macrophages as a critical responder to IL9, triggering an antimelanoma immune response.

We also demonstrated the mechanisms by which the intratumoral combination of IL9 and macrophages triggered antitumor immune responses. First, intratumoral IL9 shifted the resident macrophages at the newly developing tumor site to the M1 phenotype, thus, minimizing the protumor effects of general TAMs. Second, the intratumoral combination of TAMs and IL9 significantly increased the total number of tumor-infiltrated immune cells, which included DCs, CD103^+^ DCs, macrophages, NKs, T cells, and especially cytotoxic effector T cells. The enhancement of T- and NK-cell infiltration into solid tumors, as well as the maintenance of an adequate TME for infiltrated immune cells, are critical points for efficacious cancer immunotherapy (37, 38). Recently, intratumoral CD103^+^ DCs have been robustly reported for their critical roles in trafficking tumor antigen to lymph nodes for both direct CD8^+^ T-cell stimulation and antigen transfer to resident myeloid cells (39, 40). CD103^+^ DC deficiency resulted in the depletion of tumor-infiltrated CD8^+^ T-cell populations in melanoma (41). Collectively, our results demonstrated that IL9-polarized macrophages produced a proper TME to recruit and facilitate antitumor immune cell infiltration into the tumor mass, as well as provide an antigen-presenting cell–enriched microenvironment for the cross-presentation of tumor antigens to CD8^+^ T cells.

Next, we further elucidated how immune cells could be recruited by colocalizing IL9 and macrophages in the tumor mass. We hypothesized that *de novo* infiltrated immune cells were recruited into the TME by signals from IL9-polarized TAMs. TAMs isolated from mice bearing B16F10-IL9+BMDMs had significantly higher expression levels of chemotactic chemokines, including CCL3, CCL4, CXCL9, and CXCL10. Our observations are consistent with previous observations highlighting the role of CCL3 and CCL4 chemokine systems in recruiting antitumor CD103^+^ DCs to the tumor, especially in the tumor models that respond poorly to CPIs (27, 41–43). Similarly, the central role of CXCR3 chemokines in the tumor-targeting migration of antitumor effector T cells was also reported in adoptive T-cell transfer models (44–46). In patients with melanoma or colorectal carcinoma, the recruitment of immune cells through CXCR3/CCR5 ligands is critical for the immune-mediated rejection of tumors (47). Furthermore, tumor-targeting approaches to recruit T cells, NK cells, and CD103^+^ DCs via tumor-targeted chemokine delivery significantly improved the antitumor efficacy of CPI immunotherapy (27, 37, 38). Collectively, our results proved that the presence of TAMs at the early timepoint of tumor progression is important for the antitumor effect of IL9. The absence of macrophages at the initial time, as in the case of the B16F10-IL9–only group, or the partial depletion of TAMs during tumor progression, as in the case of treatment with clodronate, abolished the antitumor effect of IL9. The resident macrophages at the newly developing tumor site were polarized toward the M1-like phenotype by IL9. In turn, the IL9-polarized macrophages secreted chemokines, including CCL3, CCL4, CXCL9, and CXCL10, to recruit antitumor T cells, NKs, DCs, and macrophages to infiltrate the tumor. On the basis of our data, the antitumor effect of IL9 mainly depended on the surrounding immune environment, and the presence of TAMs may be essential. Our findings support the conclusion in previous studies (17–19) macrophage niches could have a decisive role in the TME, and TAM reprogramming is a promising strategy for cancer therapy.

Finally, we extended our observations using rIL9 as an antitumor therapy for macrophage-enriched tumors. Intraperitoneal treatment with rIL9 delayed tumor growth and extended the overall survival of mice bearing macrophage-enriched B16F10 melanoma and 4T1 breast cancer in a dose-dependent manner. However, the survival extension observed by intraperitoneal treatment with rIL9 was shorter than that in the intratumoral IL9 secretion model. We can infer that the amount of IL9 reaching the tumor region was small in the systemic circulation model and not evenly distributed within the tumor. Altogether, our data suggest that intratumoral supplementation with IL9 might be a viable therapeutic strategy for macrophage-enriched tumors. A very recent study on B16 melanoma metastasis showed that IL9 from CD4^+^ T cells exerted protumor functions by inducing the expression of Arg1, an M2 marker, in pulmonary interstitial macrophages (48). However, in the experimental conditions in our current study, IL9 treatment did not alter or reduced Arg1 expression at either the mRNA or protein level in subcutaneous B16F10 TAMs, BMDMs, or the RAW264.7 cell line. Furthermore, no enhancement of Arg1 expression was found in F4/80^+^ TAMs from B16F10-IL9+BMDM-coinjected mice compared with the B16F10-Mock+BMDM group. The plausible conflicting results may be attributed to the lineage diversification of macrophages, the intensity and duration of IL9 signals, and different immune cell pools of IL9 responders in the TME.

It is essential to note that until now, except for IL2 and IFNγ, the clinical use of cytokines as monotherapy has shown modest antitumor efficacy. IL15 superagonists are promising candidates in preclinical and clinical development for anticancer therapy (49). Although the remarkable antitumor efficacy of IL12 has yet to be replicated in preclinical human models, preclinical studies on the combination of IL12, IL15, and IL18 might represent ideal approaches to generating NK and DCs for cancer therapy (50, 51). Above all, combining immunostimulatory cytokines with other immunotherapeutic strategies like adoptive cell transfer or CPI has shown promising clinical data. Because we focused on clarifying the mechanism underlying the antitumor effect of IL9 in macrophage-enriched tumor models in this study, further studies are needed to develop new strategies for generating locally targeted IL9 and define the therapeutic effects of targeting IL9 in TAM-enriched cancer.

## Supplementary Material

Supplementary Figure S1 Generation of sorted bone marrow-derived macrophages (BMDMs)

Supplementary Figure S2 Treatment with IL-9 induces STAT1 and STAT3 phosphorylation in RAW 264.7 cells

Supplementary Figure S3 IL-9 promotes M1 polarization of macrophages in vitro

Supplementary Figure S4 Expression of IL-9R on the surface of B16F10 and 4T1 cells

Supplementary Figure S5 Bodyweight change in the tumor-implanted mice in Figure 3

Supplementary Figure S6 Clodronate-encapsulated liposomes show cytotoxicity to BMDMs but not to B16F10 cells

Supplementary Figure S7 FACS gating strategy used for tumor-infiltrating immune cell analysis

Supplementary Figure S8 Gating strategy to analyze the chemotactic migration of immune cells

Supplementary Figure S9 Bodyweight change of the tumor-implanted mice in Figure 6

Supplementary Table ST1 Primers used in this study
