# Supplementary figures and images for: IL9 Polarizes Macrophages to M1 and Induces the Infiltration of Antitumor Immune Cells via MIP-1 and CXCR3 Chemokines
Source: Cancer Res Commun. 2023 Jan 18;3(1):80–96. doi: 10.1158/2767-9764.CRC-22-0246 (PMC10035505; doi:10.1158/2767-9764.CRC-22-0246)

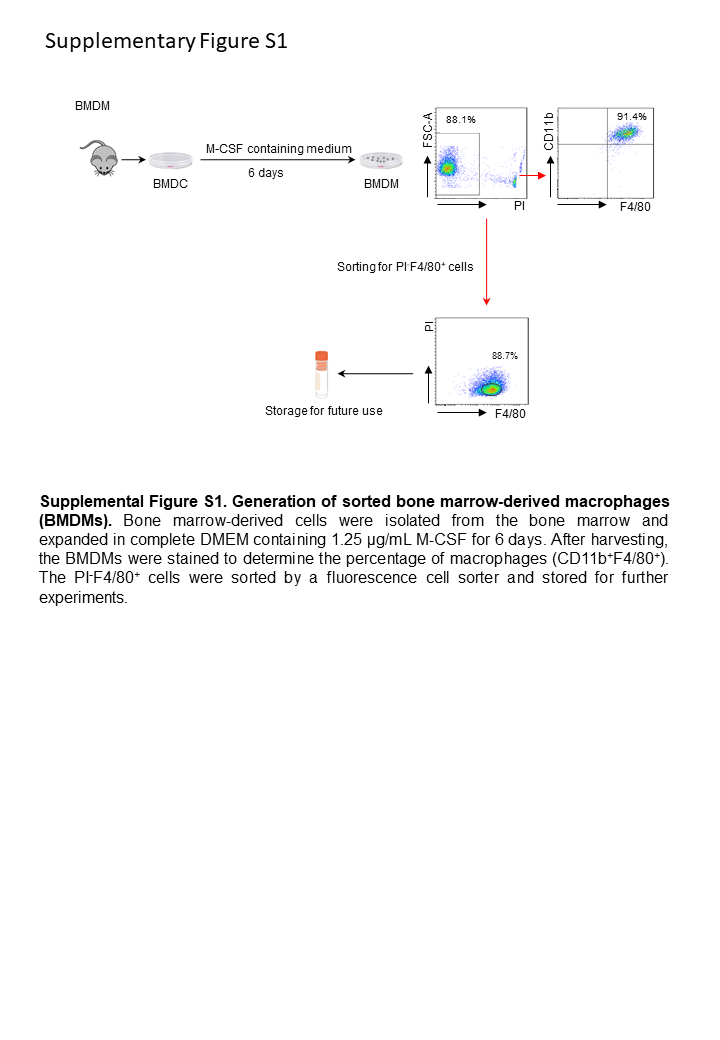

Supplement: Supplementary Figure S1 — Generation of sorted bone marrow-derived macrophages (BMDMs) [file crc-22-0246-s01.png]

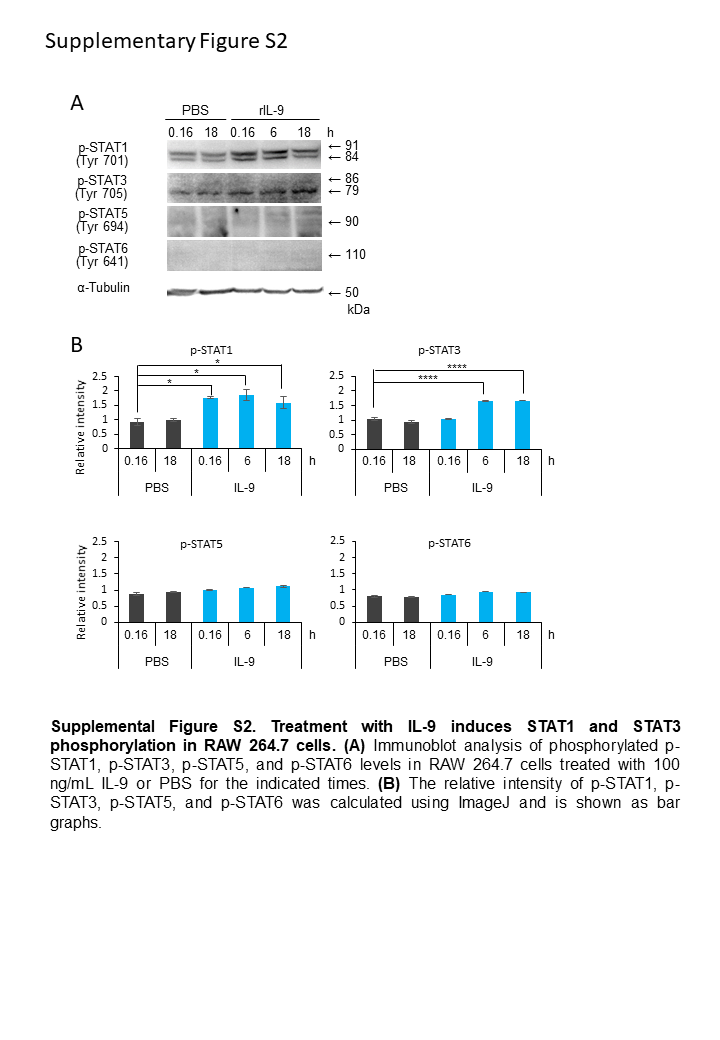

Supplement: Supplementary Figure S2 — Treatment with IL-9 induces STAT1 and STAT3 phosphorylation in RAW 264.7 cells [file crc-22-0246-s02.png]

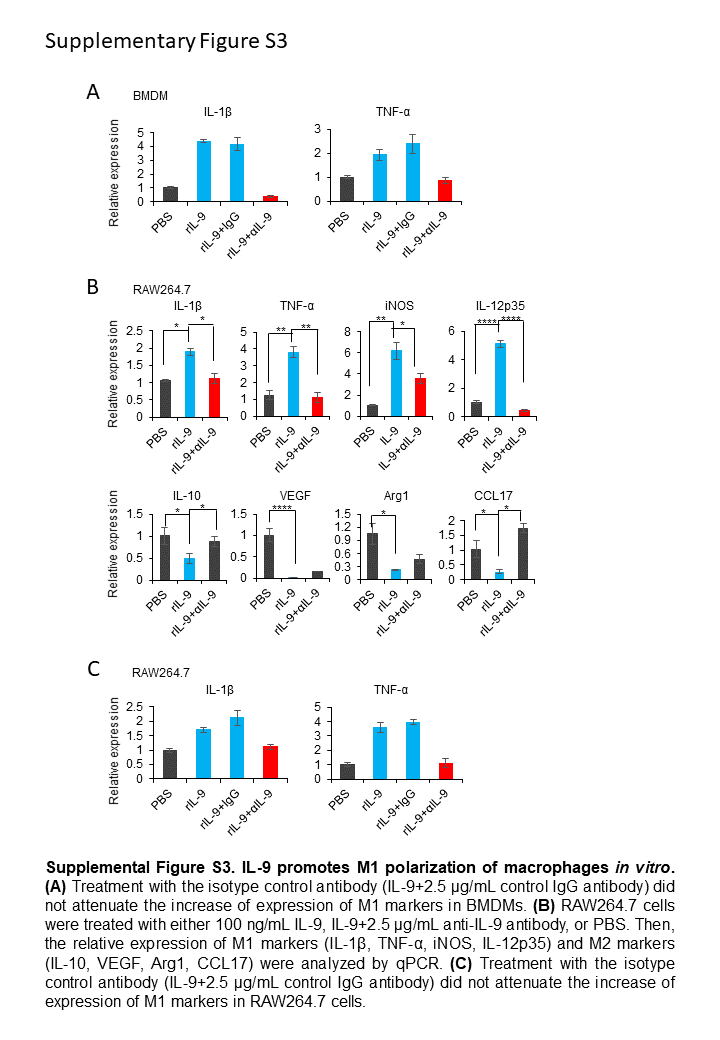

Supplement: Supplementary Figure S3 — IL-9 promotes M1 polarization of macrophages in vitro [file crc-22-0246-s03.png]

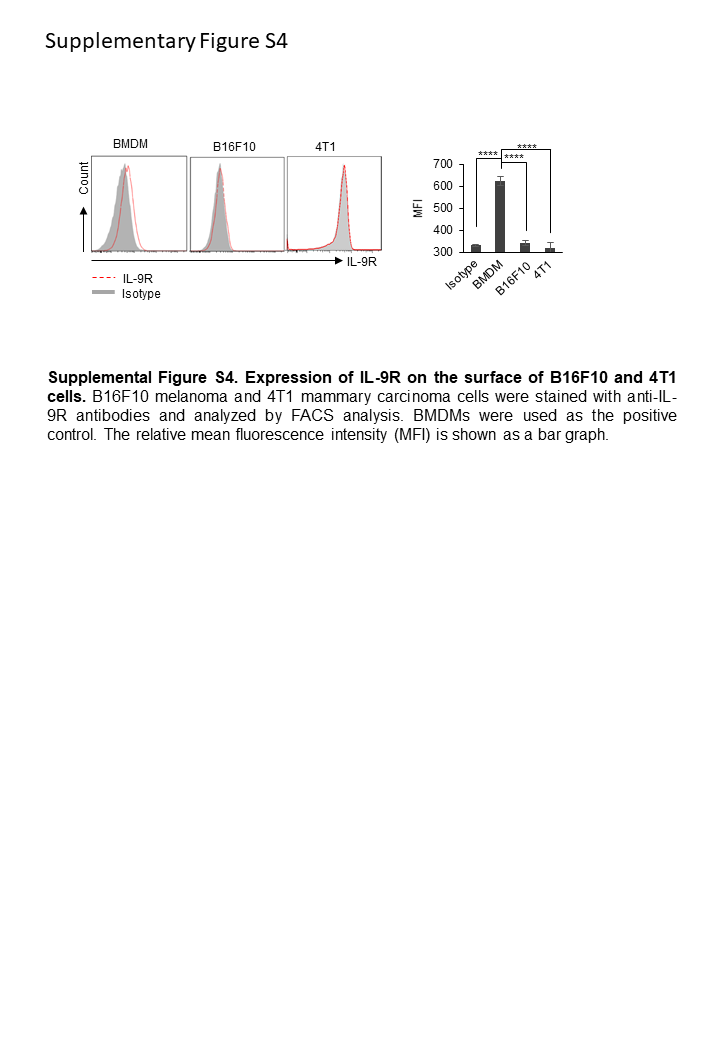

Supplement: Supplementary Figure S4 — Expression of IL-9R on the surface of B16F10 and 4T1 cells [file crc-22-0246-s04.png]

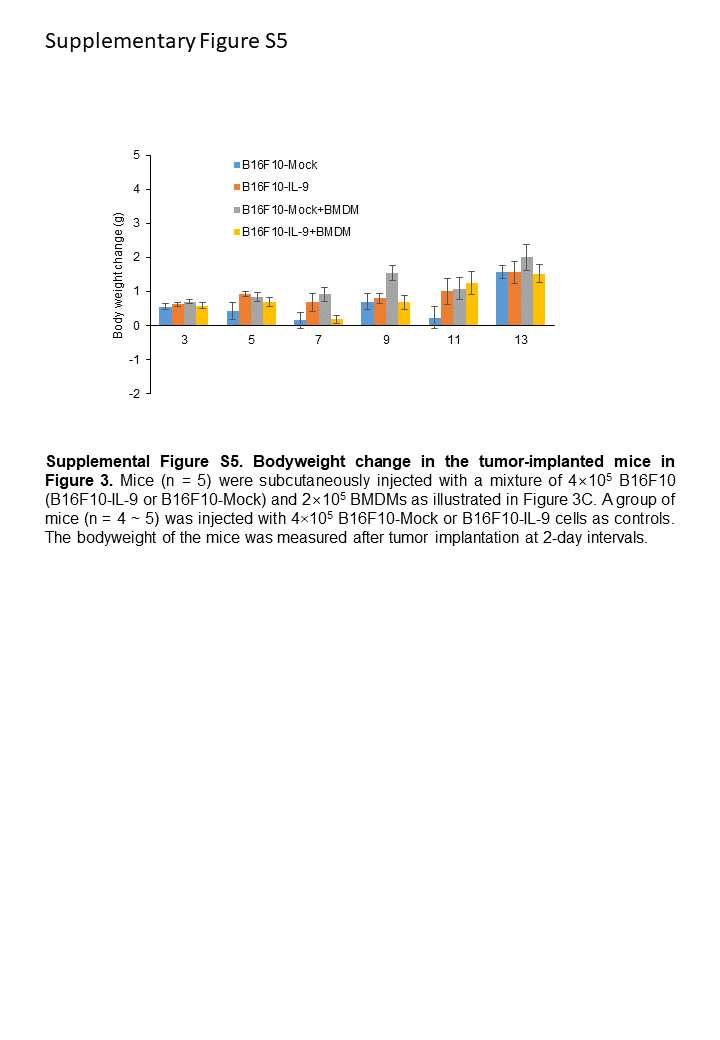

Supplement: Supplementary Figure S5 — Bodyweight change in the tumor-implanted mice in Figure 3 [file crc-22-0246-s05.png]

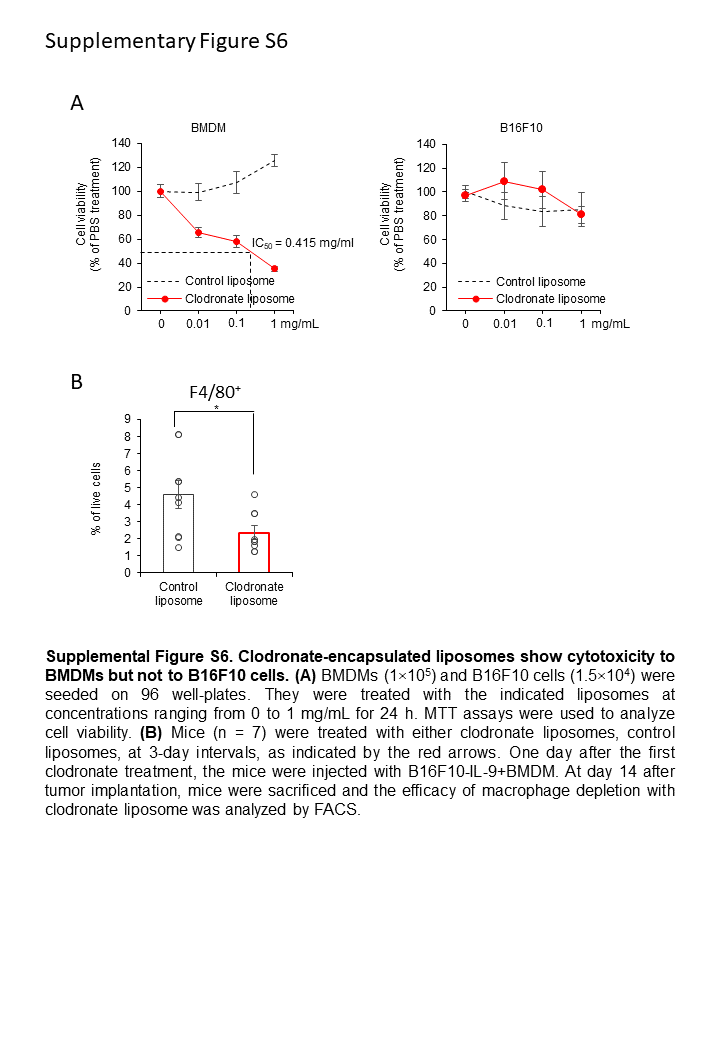

Supplement: Supplementary Figure S6 — Clodronate-encapsulated liposomes show cytotoxicity to BMDMs but not to B16F10 cells [file crc-22-0246-s06.png]

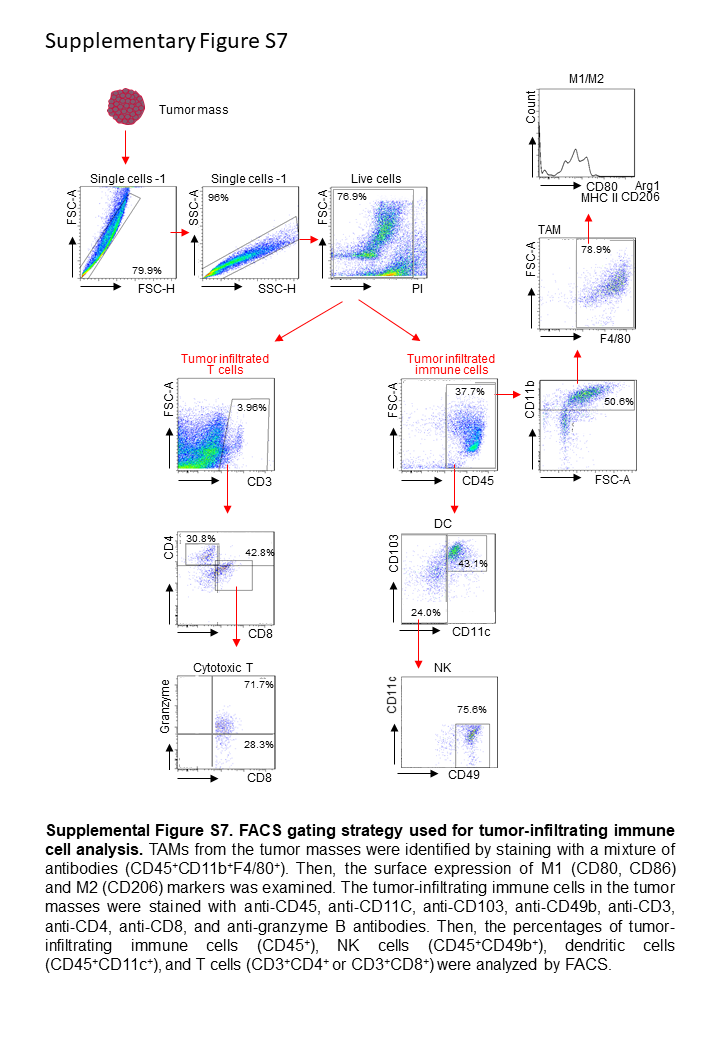

Supplement: Supplementary Figure S7 — FACS gating strategy used for tumor-infiltrating immune cell analysis [file crc-22-0246-s07.png]

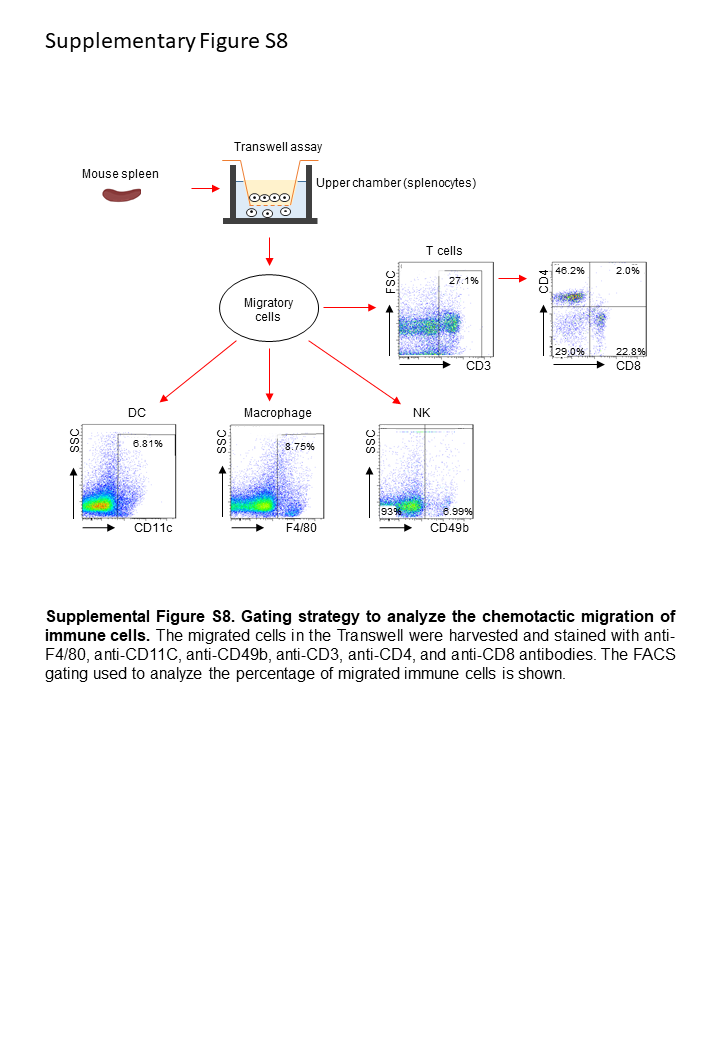

Supplement: Supplementary Figure S8 — Gating strategy to analyze the chemotactic migration of immune cells [file crc-22-0246-s08.png]

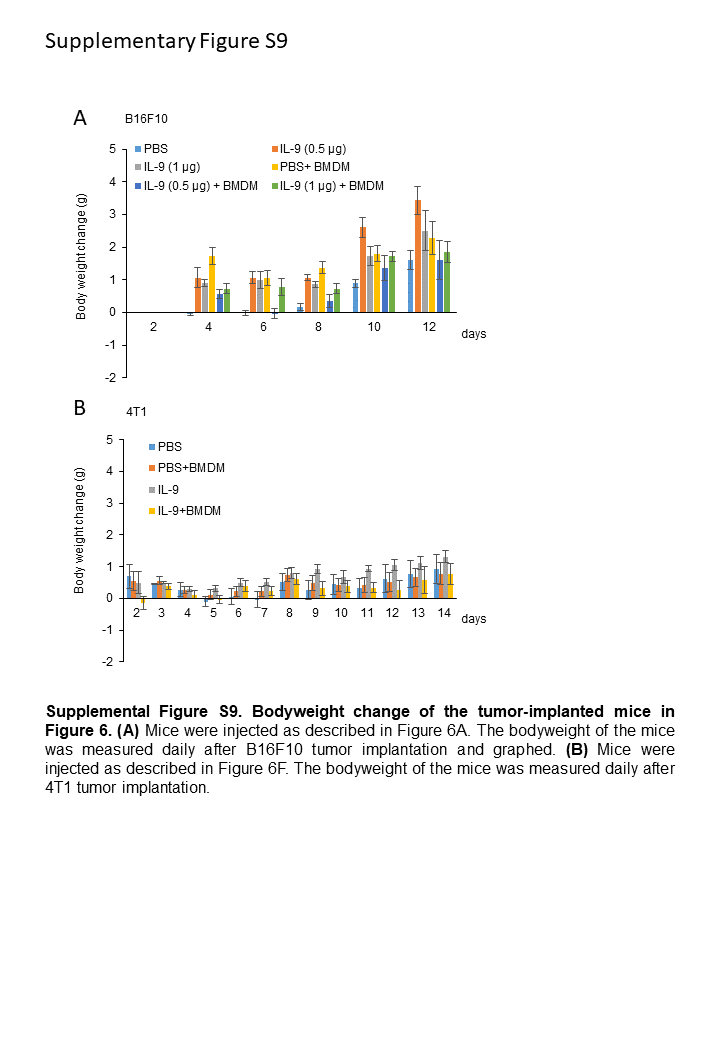

Supplement: Supplementary Figure S9 — Bodyweight change of the tumor-implanted mice in Figure 6 [file crc-22-0246-s09.png]

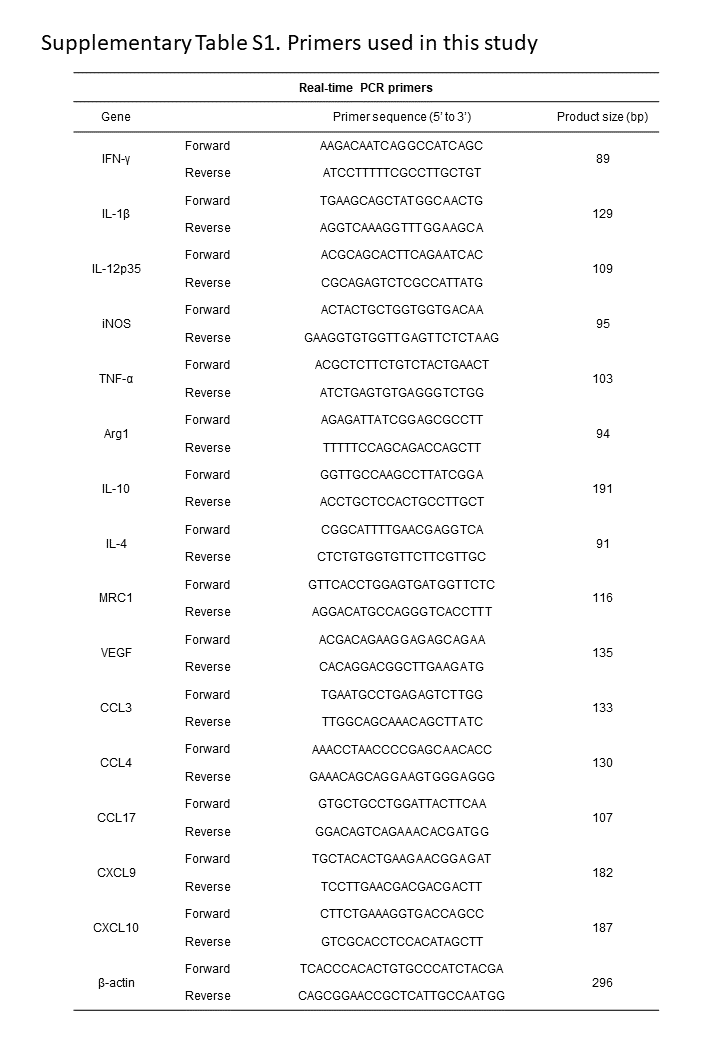

Supplement: Supplementary Table ST1 — Primers used in this study [file crc-22-0246-s10.png]
